# Essential role of glycogen synthase kinase 3 in regulating growth, drug response, and infectivity in *Leishmania infantum*

**DOI:** 10.1128/aac.00247-26

**Published:** 2026-07-06

**Authors:** Mariza Gabriela Faleiro de Moura Lodi Cruz, Fernanda Viana Moreira Silva, Giovanna Cecília de Paula e Sá, Juliana Martins Ribeiro, Giovanna Ferreira da Fonseca, Davi Alvarenga Lima, Karine Ferreira Lopes, Ana Maria Murta Santi, Silvane Maria Fonseca Murta

**Affiliations:** 1 Fundação Oswaldo Cruz-Fiocruz, Instituto René Rachou, Belo Horizonte, Minas Gerais, Brazil; 2 Fundação Oswaldo Cruz-Fiocruz, Instituto Carlos Chagas 169688 , Curitiba, Paraná, Brazil; The Children's Hospital of Philadelphia, Philadelphia, Pennsylvania, USA

**Keywords:** *Leishmania infantum*, glycogen synthase kinase 3 (GSK-3), antileishmanial drug target, drug resistance, chemotherapy

## Abstract

*Leishmania infantum* depends on tightly regulated signaling pathways to sustain growth, stress adaptation, and infectivity. Glycogen synthase kinase 3 (GSK-3) is a conserved serine/threonine kinase involved in multiple cellular processes; however, its functional role in *Leishmania* remains poorly defined. Here, we investigated the role of the short isoform of GSK-3 (GSK-3s) in *L. infantum* using a CRISPR/Cas9-based genetic approach. Attempts to generate chromosomal GSK-3s null mutants were unsuccessful, suggesting that GSK-3s is essential for parasite viability. Chromosomal gene deletion was achieved only after episomal complementation with *L. braziliensis* GSK-3s, generating a GSK3s-deficient knockdown *L. infantum* line (*LiΔchrGSK3::LbGSK3*). Quantitative PCR revealed a 3.8-fold and 9.6-fold reduction in GSK-3s transcript levels in this *L. infantum* line compared with *Li::Cas9* and GSK3-overexpressing (*Li::LbGSK3*) parasites, respectively. Reduced GSK-3s expression in this parasite line resulted in severe growth impairment, morphological alterations, and cell-cycle dysregulation. GSK-3s knockdown promastigotes exhibited a 5.3-fold increase in resistance to trivalent antimony and a 1.77-fold increase in resistance to miltefosine, as well as a striking 111-fold increase in resistance to the GSK-3 inhibitor 6-bromo-5-methylindirubin-3′-oxime (6-BIO), supporting the specificity of this compound toward parasite GSK-3s. In addition, GSK-3s-deficient parasites displayed a 7.5-fold increase in tolerance to hydrogen peroxide-induced oxidative stress, accompanied by differential modulation of antioxidant defense gene transcripts. In infection models, GSK-3s knockdown parasites displayed reduced early infectivity in THP-1-derived macrophages and significantly lower splenic parasite burdens, with reductions of 1.5-fold in BALB/c mice and 2.5-fold in IFN-*γ*^⁻/⁻^ C57BL/6 mice. Collectively, these findings suggest that GSK-3s may play an important role in regulating *L. infantum* growth, stress adaptation, drug response, and infectivity, indicating its potential as a target for anti-leishmanial drug development.

## INTRODUCTION

Leishmaniases are a group of neglected tropical diseases caused by protozoan parasites of the genus *Leishmania*, transmitted to humans and other mammals through the bite of infected female phlebotomine sand flies (1–4). The disease encompasses a broad clinical spectrum, ranging from self-limiting cutaneous lesions (cutaneous leishmaniasis, CL) to potentially fatal systemic infection (visceral leishmaniasis, VL), depending on the infecting species and the host immune response (1–4). Leishmaniasis remains a major global public health challenge, with an estimated 700,000 to 1 million new cases annually, and is endemic in 99 countries and territories worldwide, including 71 reporting both CL and VL forms (4). VL, the most severe clinical manifestation, is primarily caused by *Leishmania donovani* in Asia and Africa and by *Leishmania infantum* in the Mediterranean Basin, the Middle East, Central Asia, and the Americas, and it continues to impose a substantial public health burden in endemic regions, particularly in low- and middle-income countries (4).

In the absence of a human vaccine and given the limited effectiveness of vector control strategies, chemotherapy remains the cornerstone of leishmaniasis management (1). However, existing therapies are suboptimal. Pentavalent antimonials, amphotericin B formulations, miltefosine, paromomycin, and pentamidine constitute the current mainstay of treatment; however, their clinical use is limited by significant toxicity, prolonged and complex treatment regimens, high costs, and the increasing emergence of drug-resistant parasites (5–7). These limitations underscore the urgent need for new antileishmanial drugs with novel mechanisms of action and improved safety profiles.

Advances in parasite genomics and molecular biology have facilitated the identification of essential pathways and proteins that may serve as drug targets in *Leishmania* (8). Among these, protein kinases have emerged as particularly attractive candidates. Protein kinases are key regulators of eukaryotic cellular processes, including cell-cycle progression, differentiation, stress responses, and transcriptional control. In trypanosomatids, several kinases have been shown to be essential for parasite survival and infectivity, and their inhibition often results in profound defects in growth and infectivity (9–11). Importantly, many kinase inhibitors initially developed for human diseases have shown activity against parasite kinases, highlighting the druggability of this protein family (12).

Glycogen synthase kinase 3 (GSK-3) is a conserved serine/threonine kinase originally described as a regulator of glycogen metabolism, but now recognized as a multifunctional enzyme involved in numerous cellular processes, including cell-cycle regulation, transcription, differentiation, and stress responses (11, 13, 14). In trypanosomatids, two GSK-3 orthologs are present: a short isoform (GSK-3s) and a long isoform (GSK-3l) (15). The short isoform has been the primary target of biochemical, structural, and pharmacological studies (15). Accumulating evidence supports GSK-3s as a promising drug target in trypanosomatids (15–17). Although GSK-3 is widely conserved across diverse eukaryotes—including parasites, insects, plants, fungi, and mammals (18)—structural and comparative studies demonstrated that parasite GSK-3s exhibit differences in the ATP-binding pocket relative to the human enzyme, enabling the design of selective inhibitors with reduced host toxicity (12, 15, 19). GSK-3s has been structurally and pharmacologically characterized in other *Leishmania* species, including *Leishmania major*, *L. infantum*, and *L. donovani*, where structural analyses and inhibitor studies support its potential as a drug target (15, 17).

Despite this substantial pharmacological and structural evidence gathered for different species, direct genetic validation of GSK-3s function in *L. infantum* has been lacking. In this study, we employed a CRISPR/Cas9-based genetic approach combined with episomal complementation to investigate the role of the short isoform of GSK-3 in *L. infantum*. We assessed the impact of GSK-3s depletion on parasite growth, morphology, cell-cycle progression, susceptibility to antileishmanial drugs, tolerance to oxidative stress, and infectivity in both *in vitro* and *in vivo* models. Our findings provide genetic evidence supporting the notion that GSK-3s is required for optimal viability and infectivity of *L. infantum*, suggesting that this kinase may act as an important regulator of parasite fitness and highlighting its potential as a target for antileishmanial drug development.

## RESULTS

### Genomic localization and sequence analysis of GSK-3 in *L. infantum*

The genome of the *L. infantum* JPCM5 strain harbors two paralogous genes encoding glycogen synthase kinase 3 (GSK-3): a short isoform (GSK-3s) and a long isoform (GSK-3l). The GSK-3s is encoded by a single-copy gene (TriTrypDB accession number LINF_180007700), located on chromosome 18, spanning 1,068 bp and encoding a protein of 355 amino acids. In contrast, GSK-3l was poorly characterized. Our sequence analysis, using tBLASTn, identified LINF_220010400 as the gene corresponding to the long isoform GSK-3l (Fig. S1). This single-copy gene is located on chromosome 22, spans 2,535 bp, and encodes a predicted protein of 844 amino acids annotated as a putative protein kinase. The *L. infantum* GSK-3l shares 89.9% sequence identity and 91.8% similarity with its *L. mexicana* ortholog (LmxM.22.0490) (11). Notably, sequence alignment between the short (LINF_180007700) and long (LINF_220010400) isoforms in *L. infantum* revealed only 43.6% identity and 63.9% similarity (Fig. S2). These findings suggest limited functional redundancy and indicate that the two isoforms are unlikely to be directly compensatory.

### Generation of GSK-3s knockdown *L. infantum*

Our first attempt to delete GSK-3s in *L. infantum* was performed using the CRISPR/Cas9 system following the protocol described by Beneke and collaborators (8). Parasites were transfected with the sgRNA, 5′UTR and 3′UTR targeting sequences, and donor DNAs carrying either NEO or PURO resistance cassettes, as well as with both donor DNAs simultaneously. Only parasites transfected with the NEO donor survived neomycin selection. PCR analysis using primer pairs H and I (Table S1) confirmed the persistence of GSK-3s in these parasites, indicating that only a single allele had been disrupted. Cells transfected with both donor cassettes failed to survive. After three unsuccessful attempts to generate double-knockout mutants, our results suggest that GSK-3s is likely an essential gene for parasite survival.

We next adopted a rescue strategy to enable chromosomal deletion by providing an episomal copy of GSK-3s. An expression construct containing the GSK-3s gene from *L. braziliensis* (MHOM/BR/75/M2903) was generated by cloning the coding sequence into the pIR1-SAT plasmid using the Gibson Assembly method. Correct assembly and sequence integrity of pIR1SAT_LbGSK3 were confirmed by Sanger sequencing with an M13 primer.

To assess the suitability of *L. braziliensis* GSK-3s for episomal complementation, a global protein sequence alignment was performed using the MAFFT tool, comparing GSK-3s from *L. infantum* (LINF_180007700) and *L. braziliensis* (LBRM2903_180007900). The alignment, spanning 355 amino acids, revealed a high degree of conservation between the two proteins, with 93.5% sequence identity and 96.9% similarity (Fig. S3).

**Fig 1:**
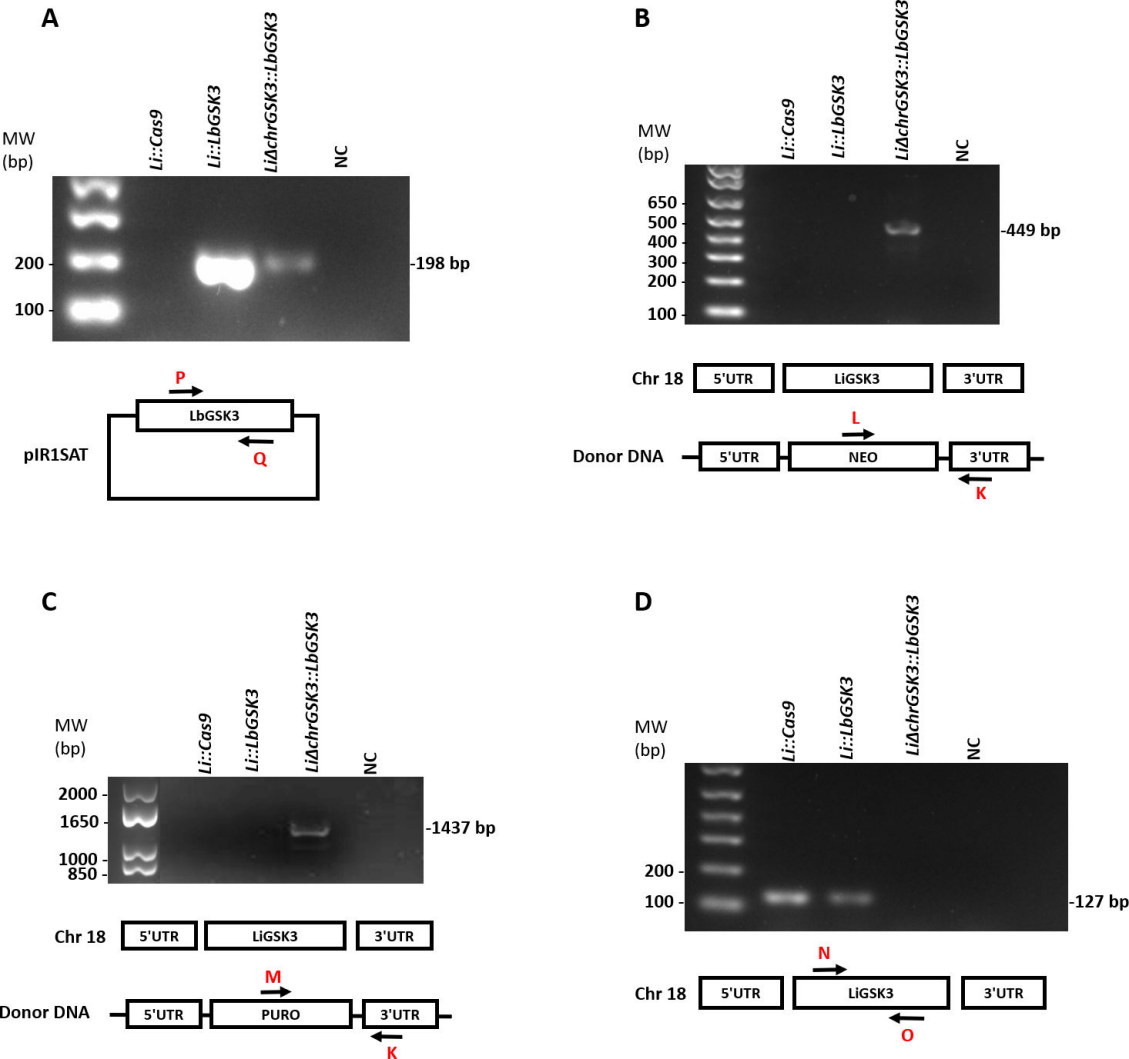
Chromosomal GSK-3s knockout in *L. infantum* expressing episomal *L. braziliensis* GSK-3s. First, the knockout was assessed by PCR using genomic DNA from *Li::Cas9* and mutant parasites. (**A**) Confirmation of the presence of the episomal *L. braziliensis* GSK-3s gene. The correct integration of the (**B**) NEO and (**C**) PURO resistance markers was evaluated by PCR using one primer annealing to the 3′ UTR region of GSK-3 and another primer annealing to the resistance marker sequence. (**D**) Confirmation of the parasites knocked out for the chromosomal *L. infantum* GSK-3s gene. Please note that the schematic representations of the molecules are not to scale, with sizes and distances adjusted for clarity rather than accuracy. MW: molecular weight standard; bp: base pairs; NC: negative control.

Then, *L. infantum* parasites constitutively expressing *Sp*Cas9 and T7 RNA polymerase (20), as well as the red fluorescent protein *tdTomato* (21), were transfected with the resulting pIR1SAT_LbGSK3 plasmid, generating the mutant parasite line *Li::LbGSK3*. PCR analysis confirmed the presence of episomal *LbGSK3* in the transfected parasites (Fig. 1A).

In the second knockout attempt, parasites *Li::LbGSK3* expressing *Sp*Cas9, *tdTomato,* and exogenous *LbGSK3* were transfected with donor DNAs and gRNA templates to generate GSK-3s chromosomal null mutants. In contrast to the initial attempts, parasites harboring both donor cassettes survived under combined neomycin and puromycin selection, indicating that the episomal copy successfully complemented the essential function of GSK-3s.

Then, the *LiΔchrGSK3::LbGSK3* mutant clone was subjected to PCR to verify the integration of the donor DNAs into the alleles containing the GSK-3s gene. The correct integration of the NEO and PURO molecular markers was confirmed with specific primers for the 3′UTR region of the GSK-3s gene adjacent to the donor DNA (primer K) and the coding sequence of NEO or PURO (primers L or M), generating fragments of 449 bp and 1,437 bp, respectively, in the mutant line (Fig. 1B and C). The absence of amplification of the chromosomal *L. infantum* GSK-3s using primers N and O (Table S1) confirmed complete deletion of the endogenous locus in the *LiΔchrGSK3::LbGSK3* line (Fig. 1D).

**Fig 2:**
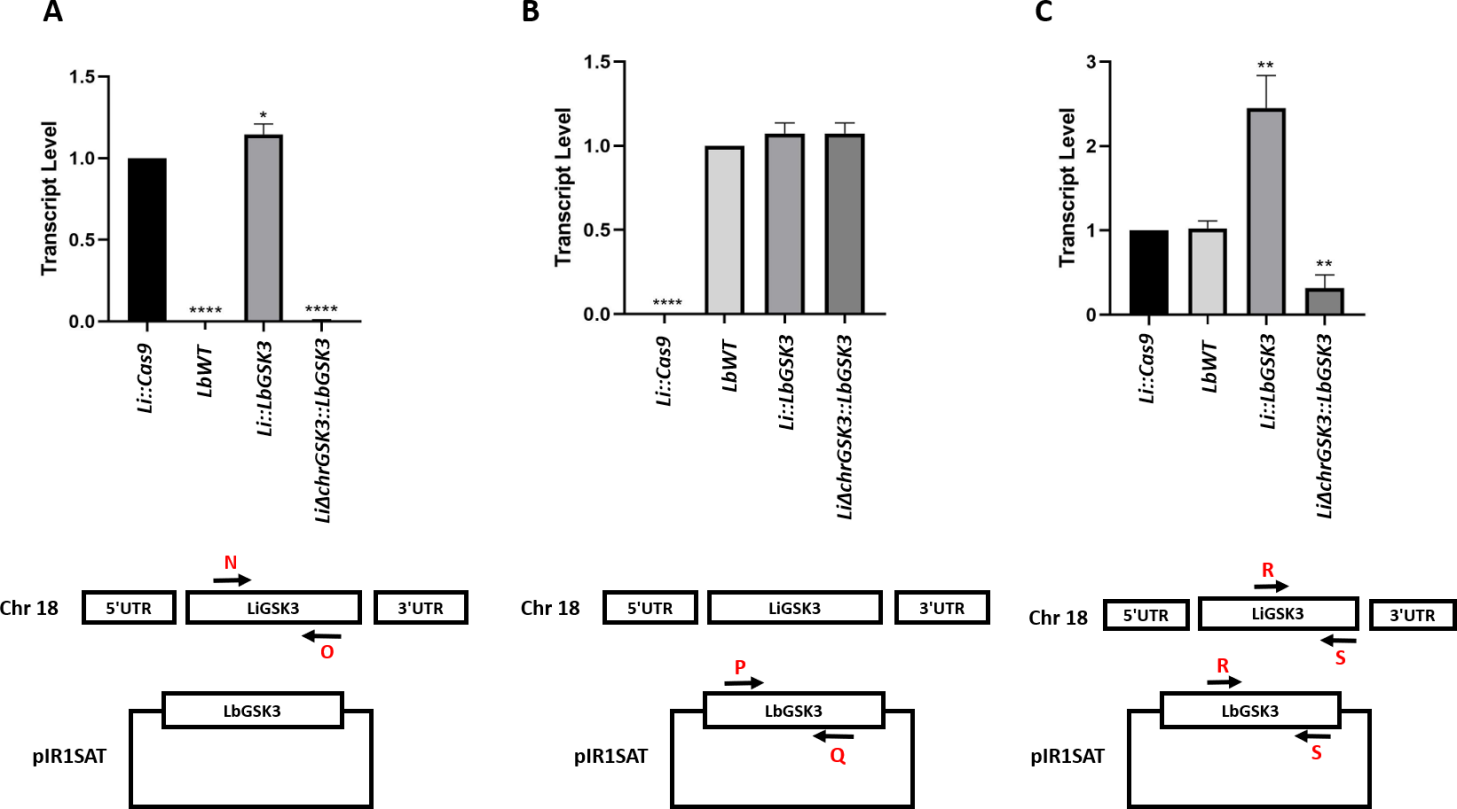
Transcription levels of GSK-3s were assessed by RT-qPCR in *L. infantum* mutant parasites. (**A**) Transcription levels of the *LiGSK3* gene in *Li::Cas9* and mutant promastigotes parasites were assessed using a pair of primers that only amplify *LiGSK3*. (**B**) Transcription levels of the *LbGSK3* gene in *Li::Cas9* and mutant parasites were assessed using a pair of primers that only amplify *LbGSK3*. (**C**) Transcription levels of both *LiGSK3* and *LbGSK3* in *Li::Cas9* and mutant parasites were assessed using a pair of primers that recognize both sequences. DNA polymerase gene (LINF_160021500) was used as a constitutive normalizer, and the fold-change was calculated by the 2^-ΔΔCT^ comparative method. One-way analysis of variance with Dunnett post hoc test was used to compare the control parasites and mutants. We used *Li::Cas9* as the control. ^*^P < 0.05, ^**^P < 0.01, ^***^P < 0.001, and ^****^P < 0.0001.

RT-qPCR was used to differentiate between chromosomal *LiGSK-3s* (original GSK-3s copies from *L. infantum*) and episomal *LbGSK-3s* (ectopic copies from *L. braziliensis*). Using specific primers for GSK-3s in *L. infantum* (primers N and O), the GSK-3s transcript level was only observed in the *Li::Cas9* line and *Li::LbGSK3* parasites (Fig. 2A). No transcript level of endogenous GSK-3s was detected in the *LbWT* and *LiΔchrGSK3::LbGSK3* (Fig. 2A). In contrast, using specific primers for *LbGSK3* (primers P and Q, Table S1), GSK-3s transcript levels were observed in all lines tested, except for the *Li::Cas9* line, which did not contain the pIR1SAT_LbGSK3 plasmid (Fig. 2B). This suggests that deletion of endogenous GSK-3s in *L. infantum* lines was only possible because of the presence of *LbGSK3* in the episomal form.

We next quantified total GSK-3s expression using primers designed to amplify conserved regions shared between both *Leishmania* species (primers R and S). Parasites expressing exogenous *L. braziliensis* GSK-3 (*Li::LbGSK3*) exhibited a 2.7-fold increase (^**^P < 0.01) in GSK-3s transcript levels compared with *Li::Cas9* and *LbWT* parasites. In contrast, GSK-3s transcript abundance was markedly reduced in *LiΔchrGSK3::LbGSK3* parasites (Fig. 2C). Specifically, GSK-3s expression in this mutant was decreased by 3.8-fold and 9.6-fold (^**^P < 0.01) relative to *Li::Cas9* and *Li::LbGSK3* parasites, respectively.

To investigate whether the long GSK-3 isoform of *L. infantum* (LINF_220010400) could compensate for the reduced expression of GSK-3s isoform in *LiΔchrGSK3::LbGSK3* parasites, the transcript levels of GSK-3l were quantified by qPCR (Fig. S4). The analysis revealed no significant differences in transcript levels when compared to the *Li::Cas9* control line, indicating that GSK-3l expression is not transcriptionally upregulated in response to GSK-3s knockdown in *LiΔchrGSK3::LbGSK3* parasites.

### Reduced GSK-3s expression impairs growth and alters parasite morphology in *L. infantum*

**Fig 3:**
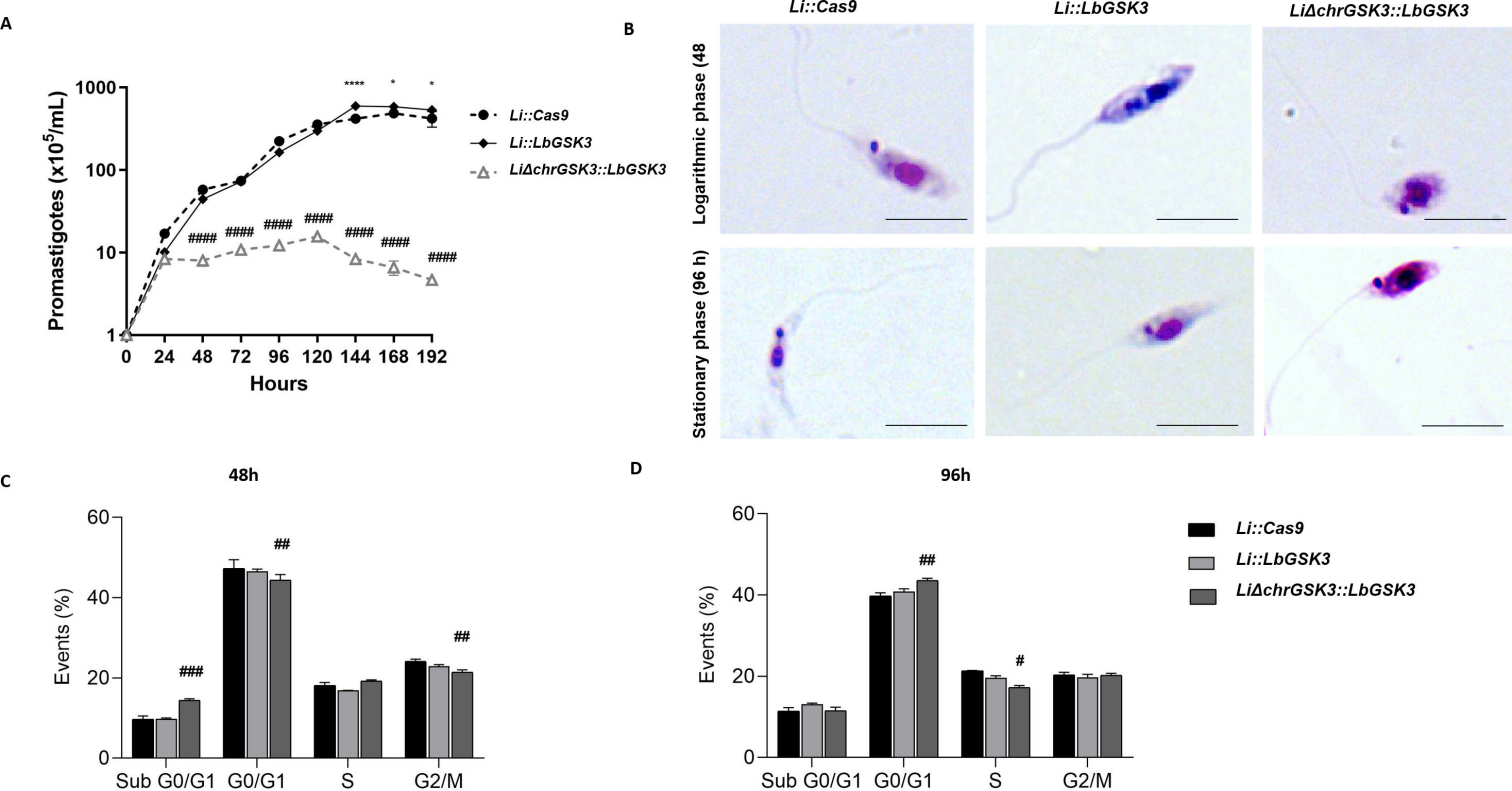
GSK-3s knockdown parasites exhibit reduced growth in culture and altered morphology compared to *Li::Cas9* parasites. (**A**) Growth curves of *Li::Cas9*, *Li::LbGSK3*, and *LiΔchrGSK3::LbGSK3* promastigotes. Parasites were seeded at 1 × 10^5^/mL and cultivated for 8 days, with cell densities determined daily. Data represent averages of three independent experiments performed in triplicate. Curves were statistically analyzed using two-way analysis of variance with Bonferroni post hoc tests. (**B**) Optical microscopy of *Li::Cas9*, *Li::LbGSK3*, and *LiΔchrGSK3::LbGSK3* promastigotes stained with Giemsa 48 h and 96 h post-passage. Images were acquired using an Axio Observer A1 fluorescence microscope (Zeiss). Scale bars: 5 μm. Cell cycle analysis of *Li::Cas9*, *Li::LbGSK3*, and *LiΔchrGSK3::LbGSK3* at 48 (**C**) and 96 (**D**) h of growth, respectively. The parasites were stained with propidium iodide and evaluated by flow cytometry. Data represent the mean ± standard deviation of three independent experiments performed in triplicates. We used *Li::Cas9* as the control. ^*^P < 0.05, ^**^P < 0.01, ^***^P < 0.001, and ^****^P < 0.0001 for *Li::Cas9 vs. Li::LbGSK3*; ^#^*P* < 0.05, ^##^*P* < 0.01, ^###^*P* < 0.001, ^####^*P* < 0.0001 for *Li::Cas9 vs. LiΔchrGSK3::LbGSK3*.

We assessed the *in vitro* growth of mutant and *Li::Cas9* parasites over an 8-day period (192 h) (Fig. 3A). *LiΔchrGSK3::LbGSK3* parasites exhibited a pronounced growth defect, showing significantly reduced proliferation at all evaluated time points compared with both control lines (*Li::Cas9* and *Li::LbGSK3*) (^####^*P* < 0.0001). These results indicate that the reduced GSK-3s expression compromises the parasites’ capacity to sustain normal growth, even in the presence of an episomal copy.

The GSK-3S knockdown mutant line (*LiΔchrGSK3::LbGSK3*) exhibited marked morphological alterations, including reduced cell body size and abnormal morphology at both 48 h and 96 h of growth compared with control parasites (Fig. 3B; scale bar, 5 μm), supporting an essential role for the chromosomal GSK-3s gene in maintaining parasite structural integrity and normal growth dynamics. Morphometric analysis demonstrated significant differences in both cell body size and flagellum length among the parasite lines analyzed. At 48 h of growth, *Li::Cas9* and *Li::LbGSK3* parasites displayed comparable cell body sizes (5.61 ± 0.55 µm and 5.32 ± 0.82 µm, respectively), whereas *LiΔchrGSK3::LbGSK3* parasites exhibited a significantly smaller cell body (4.13 ± 0.75 µm; ^####^*P* < 0.0001). At 96 h, the cell body size increased in *Li::Cas9* (6.58 ± 0.82 µm) and *Li::LbGSK3* parasites (6.94 ± 0.87 µm), while *LiΔchrGSK3::LbGSK3* parasites remained significantly smaller (4.28 ± 0.49 µm; ^####^*P* < 0.0001). Flagellum length was similar among all parasite lines at 48 h of growth (*Li::Cas9*: 5.45 ± 1.83 µm; *Li::LbGSK3*: 5.01 ± 1.63 µm; *LiΔchrGSK3::LbGSK3*: 5.77 ± 1.72 µm). However, at 96 h, *LiΔchrGSK3::LbGSK3* parasites exhibited a markedly elongated flagellum (10.46 ± 1.79 µm; ^####^*P* < 0.0001) compared with *Li::Cas9* (5.62 ± 1.79 µm) and *Li::LbGSK3* parasites (6.78 ± 1.57 µm).

Cell cycle analysis by flow cytometry following propidium iodide staining revealed time-dependent alterations in GSK-3s knockdown parasites compared with *Li::Cas9* control parasites indicative of apoptosis. At 48 h (Fig. 3C), *LiΔchrGSK3::LbGSK3* parasites exhibited a significant increase in the Sub G0/G1 population (^###^*P* < 0.001). The peak in Sub G0/G1 population represents a population of cells with hypodiploid DNA content, a hallmark of apoptosis resulting from DNA fragmentation during programmed cell death (22). As cells undergo apoptosis, G0/G1 and G2/M populations shrink (^##^*P* < 0.01). At 96 h (Fig. 3D), *LiΔchrGSK3::LbGSK3* parasites maintained a dysregulated cell cycle profile, characterized by a relative accumulation in the G0/G1 phase (^##^*P* < 0.01) and a decrease in the S-phase population (^#^*P* < 0.05). This accumulation in the G0/G1 phase corresponds to a diploid (2N DNA) population of cells in either quiescence (G0) or pre-S phase (G1). Notably, G0 represents a quiescent, non-dividing, but viable state with diploid DNA content (23). No significant alterations in cell cycle distribution were observed in *Li::LbGSK3* parasites at 48 or 96 h. The representative histograms are shown in Fig. S5.

### GSK-3s knockdown parasites are more resistant to Sb^III^, miltefosine, and indirubin derivatives

**Fig 4:**
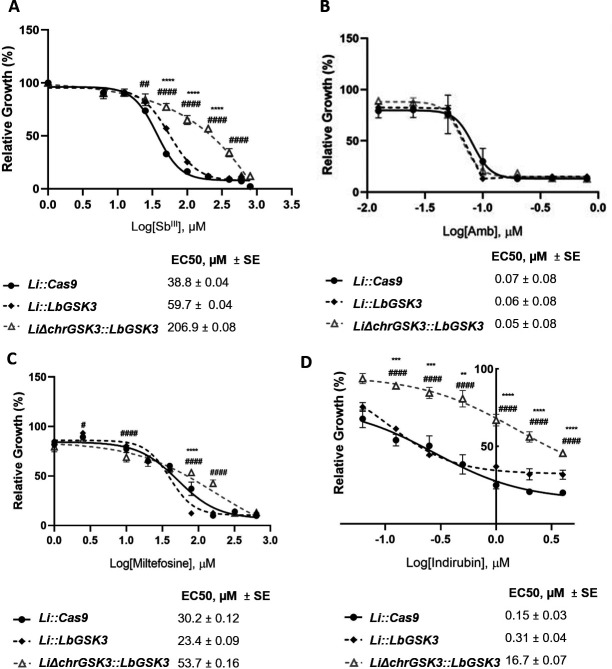
Drug susceptibility profiles of *L. infantum* promastigotes expressing altered GSK-3s levels. *Li::Cas9* and GSK-3s mutant promastigote parasites were cultured in the presence of different concentrations of (**A**) Sb^III^ (6.25–800 μM), (**B**) amphotericin B (0.0125–0.8 μM), (**C**) miltefosine (5–640 μM), and (**D**) 6-bromo-5-methylindirubin-3′-oxime (6-BIO) (0.0625–20 μM). Parasite growth was determined after 48 h of incubation with or without the drug. Data plotted in the dose–response curve represent the mean with standard deviations of three independent experiments performed in triplicate. The EC_50_ was determined using a non-linear regression variable-slope model with the ‘log (inhibitor) *versus* response’ equation in GraphPad Prism. Two-way analysis of variance with Dunnett post hoc test was used to compare *Li::Cas9* parasites and mutants for each drug concentration; ^*^P < 0.05, ^**^P < 0.01, ^***^P < 0.001, and ^****^P < 0.0001 for *Li::Cas9* vs. *li::LbGSK*3; ^##^*P* < 0.01, ^###^*P* < 0.001, and ^####^*P* < 0.0001 for *Li::Cas9 vs. LiΔchrGSK3::LbGSK3*.

We next evaluated whether reduced GSK-3s expression in *L. infantum* mutant line affected parasite susceptibility to leishmanicidal drugs. The promastigote forms of the *LiΔchrGSK3::LbGSK3* mutant line exhibited a 5.3-fold increase (^####^*P* < 0.0001) in resistance to Sb^III^ compared with the *Li::Cas9* control, with EC_50_ values of 206.9 ± 0.08 µM and 38.8 ± 0.04 µM, respectively (Fig. 4A). In addition, *Li::LbGSK3* parasites displayed a 1.5-fold increase (^***^P < 0.001) in Sb^III^ resistance relative to *Li::Cas9* (EC_50_ = 59.7 ± 0.04 µM). In contrast, susceptibility to amphotericin B did not differ significantly among the analyzed lines (Fig. 4B), with EC_50_ values ranging from 0.05 to 0.07 μM. Miltefosine susceptibility was also influenced by reduced GSK-3s expression (Fig. 4C), as *LiΔchrGSK3::LbGSK3* parasites showed a 1.77-fold increase (^###^*P* < 0.001) in resistance compared with *Li::Cas9* (EC_50_ = 53.7 ± 0.16 µM *versus* 30.2 ± 0.12 µM). In contrast, parasites overexpressing GSK-3s (*Li::LbGSK3*) showed increased susceptibility to miltefosine, with a 1.3-fold decrease (^***^P < 0.001) in resistance relative to *Li::Cas9* (EC₅₀ = 23.4 ± 0.09 µM).

Additionally, we evaluated drug susceptibility in intracellular amastigotes using THP-1–derived macrophages infected with the *L. infantum* lines (Fig. S6). Interestingly, the susceptibility profiles observed in both parasite promastigote and intracellular amastigote stages were highly consistent. For Sb^III^ treatment, the *Li::Cas9* control line displayed an EC_50_ of 3.75 ± 0.61 µM, while *Li::LbGSK3* parasites showed a comparable value (3.63 ± 0.53 µM) (Fig. S6A). In contrast, *LiΔchrGSK3::LbGSK3* parasites exhibited a significantly higher EC_50_ of 19.84 ± 0.94 µM, corresponding to a 5.2-fold increase in resistance compared with the control line (^####^*P* < 0.0001). Similarly, for miltefosine, the EC_50_ values were 4.03 ± 0.06 µM for *Li::Cas9*, 2.83 ± 0.04 µM for *Li::LbGSK3*, and 7.00 ± 0.05 µM for *LiΔGSK3::LbGSK3*, representing a 1.7-fold increase in resistance relative to the control parasites (^##^*P* < 0.01) (Fig. S6B).

A striking phenotype was observed upon treatment with the indirubin derivative 6-bromo-5-methylindirubin-3′-oxime (6-BIO) (Fig. 4D). *LiΔchrGSK3::LbGSK3* knockdown parasites exhibited a 111-fold increase (^####^*P* < 0.0001) in resistance compared with *Li::Cas9*, with EC_50_ values of 16.7 ± 0.07 µM and 0.15 ± 0.03 µM, respectively. This pronounced loss of sensitivity strongly supports the specificity of 6-BIO for GSK-3s in *L. infantum*, consistent with its established mechanism of action as a GSK-3s inhibitor. In addition, *Li::LbGSK3* parasites displayed a 2.0-fold increase (^***^P < 0.001) in 6-BIO resistance relative to *Li::Cas9* (EC_50_ = 0.31 ± 0.04 µM).

### Reduced GSK-3s expression enhances oxidative stress tolerance and modulates antioxidant defense gene expression in *L. infantum*

**Fig 5:**
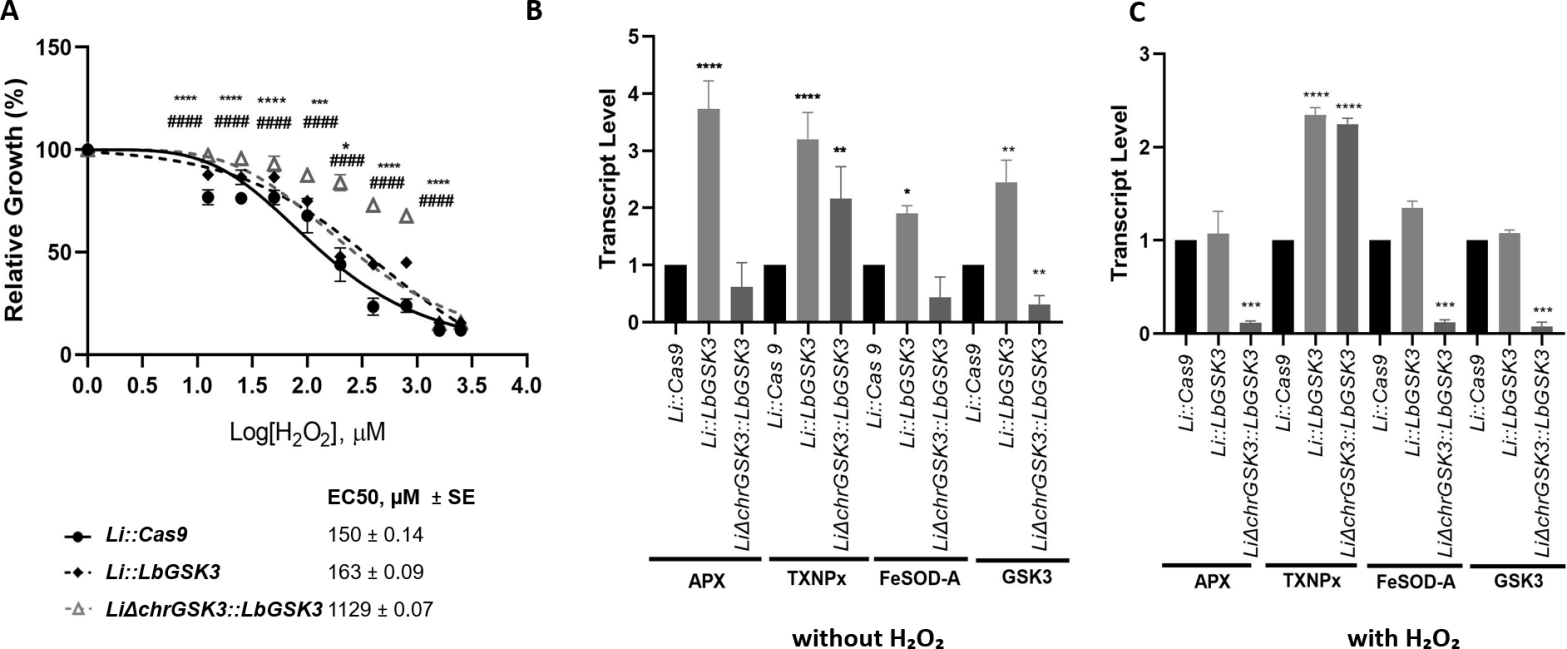
Reduced GSK-3s expression confers oxidative stress tolerance and appears to modulate antioxidant defense system enzymes. *Li::Cas9* and mutant promastigote parasites were cultured in the presence of different concentrations of (**A**) H₂O₂ (12.5–2,500 μM). The EC_50_ was determined using a non-linear regression variable-slope model with the “log(inhibitor) *versus* response” equation in GraphPad Prism. Transcription levels of ascorbate peroxidase (*APX*), tryparedoxin peroxidase (*TXNPx*), iron superoxide dismutase A (*FeSODA*), and glycogen synthase kinase 3 short (GSK-3s) were assessed by RT-qPCR in *Li::Cas9* and mutant parasites without H_2_O_2_ (**B**) and with H_2_O_2_ (**C**). Two-way analysis of variance (ANOVA) with Dunnett’s post hoc test was used to compare *Li::Cas9* parasites and mutants for each drug concentration. DNA polymerase gene was used as a constitutive normalizer, and the fold-change was calculated by the 2 ^–∆∆Ct^ method. One-way ANOVA with Dunnett’s post hoc test was used to compare control parasites and mutants. *Li::Cas9* was used as the control. ^*^P < 0.05, ^**^P < 0.01, ^***^P < 0.001, and ^****^P < 0.0001 for *Li::Cas9 vs. Li::LbGSK3*; ^###^*P* < 0.001 and ^####^*P* < 0.0001 for *Li::Cas9 vs. LiΔchrGSK3::LbGSK3*.

We assessed the tolerance of promastigote and intracellular amastigote forms of *L. infantum* mutant parasites to oxidative stress induced by H₂O₂. In promastigotes, *LiΔchrGSK3::LbGSK3* knockdown parasites exhibited a 7.5-fold increase in tolerance to H₂O₂ compared with *Li::Cas9* parasites (^####^*P* < 0.0001), with EC_50_ values of 1,129 ± 0.07 µM and 150 ± 0.14 µM, respectively (Fig. 5A). A similar phenotype was observed in intracellular amastigotes (Fig. S6C). The *Li::Cas9* control line presented an EC_50_ of 12.52 ± 0.30 µM, whereas *Li::LbGSK3* parasites exhibited an increased EC_50_ of 19.92 ± 0.40 µM, corresponding to a 1.6-fold increase in tolerance (^**^P < 0.01). Notably, *LiΔchrGSK3::LbGSK3* parasites displayed the highest EC_50_ value (43.81 ± 0.55 µM), representing a 3.5-fold increase in tolerance relative to the control line (^####^*P* < 0.0001).

Given the enhanced tolerance to oxidative stress observed in GSK-3s knockdown parasites, we hypothesized that compensatory regulation of antioxidant defense genes might contribute to this phenotype. To investigate this possibility, transcript levels of ascorbate peroxidase (*APX*), tryparedoxin peroxidase (*TXNPx*), and iron superoxide dismutase A (*FeSODA*) were quantified by RT-qPCR in promastigote forms of the *L. infantum* mutant lines (Fig. 5B). Parasites overexpressing GSK-3s (*Li::LbGSK3)* exhibited marked upregulation of antioxidant defense genes, with increases of 4.7-fold, 4.16-fold (^****^P < 0.0001), and 3.2-fold (^*^P < 0.05) in *APX, TXNPx,* and *FeSODA* transcript levels, respectively, compared with *Li::Cas9* parasites. In contrast, *LiΔchrGSK3::LbGSK3* knockdown parasites showed a selective 2.77-fold increase (^**^P < 0.01) in *TXNPx* transcripts, with no significant alterations in *APX* or *FeSODA*. Upon exposure to H_2_O_2_, distinct antioxidant gene expression profiles were observed among the *L. infantum* parasite lines (Fig. 5C). As expected, *LiΔchrGSK3::LbGSK3* parasites maintained reduced GSK-3s transcript levels regardless of oxidative stress conditions. Under H_2_O_2_ treatment, these parasites exhibited a marked reduction in *APX* (7-fold, ^***^P < 0.001) and *FeSODA* (10-fold, ^***^ *P* < 0.001) transcript levels compared with *Li::Cas9* parasites. In contrast, *TXNPx* transcripts remained consistently elevated in this knockdown line both in the absence (2.77-fold, ^**^P < 0.01) and presence (2.2-fold, ^****^P < 0.0001) of H_2_O_2_ treatment, suggesting that selective upregulation of tryparedoxin peroxidase may represent a compensatory adaptive mechanism associated with oxidative stress tolerance in GSK-3s-deficient parasites.

Interestingly, although *Li::LbGSK3* parasites displayed a 2.7-fold increase in GSK-3s transcript levels under basal conditions, exposure to H_2_O_2_ reduced GSK-3s expression to levels comparable to those observed in *Li::Cas9* parasites (Fig. 5C). Consistent with this reduction, *APX* and *FeSODA* transcript levels also became similar to those of the control line under oxidative stress conditions. Nevertheless, despite the decrease in GSK-3s transcript abundance, *Li::LbGSK3* parasites maintained significantly elevated *TXNPx* expression both in the absence (4.16-fold, ^****^P < 0.0001) and presence (2.3-fold, ^****^P < 0.0001) of H₂O₂ treatment. Together, these findings suggest that *TXNPx* expression is particularly responsive to alterations in GSK-3s levels and may play an important role in the adaptive oxidative stress response of *L. infantum*.

### GSK-3s knockdown reduces early macrophage infection and parasite burden *in vivo*

**Fig 6:**
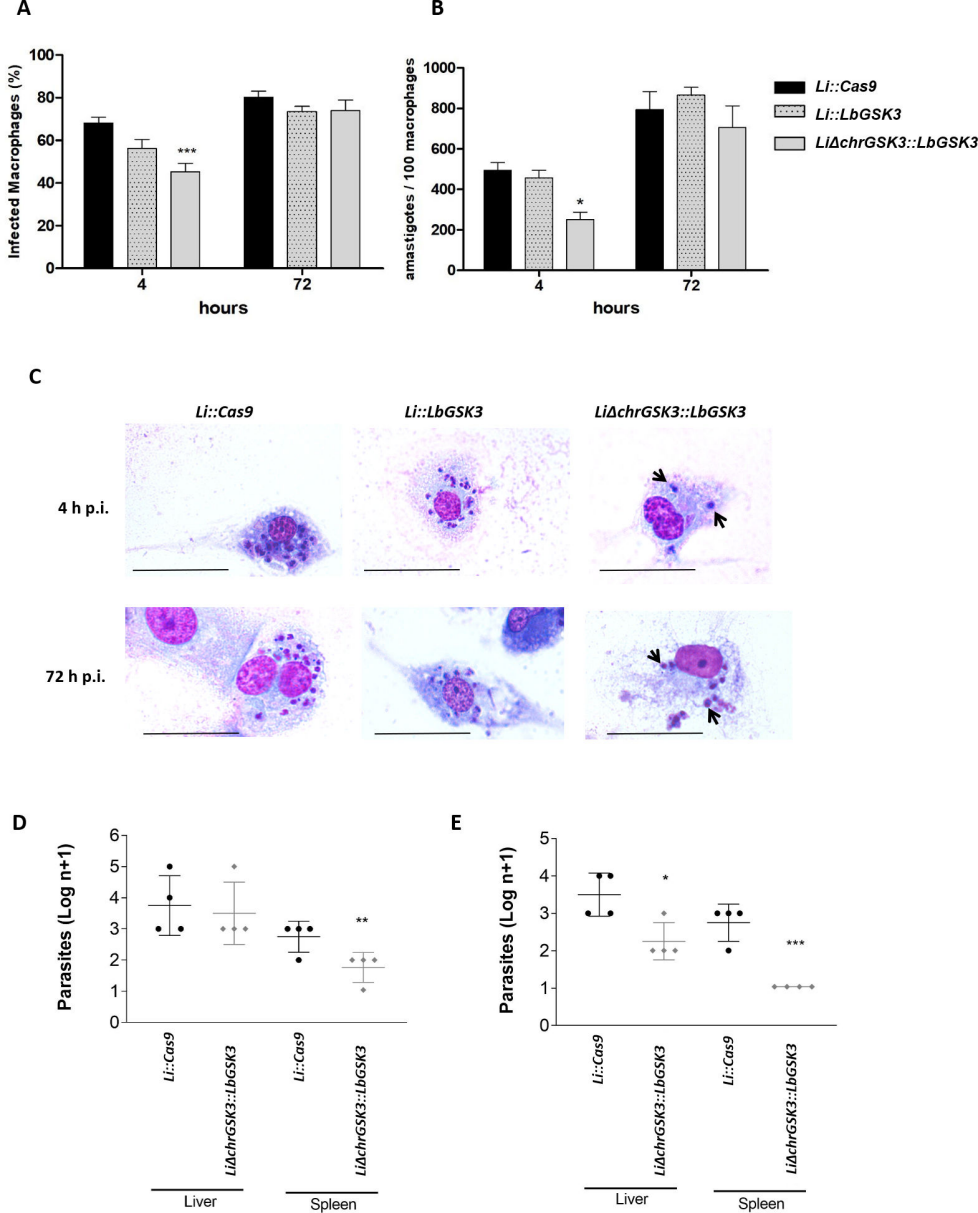
Impact of GSK-3s knockdown on *L. infantum* infectivity in macrophages and mice. Evaluation of *Li::Cas9* and mutant (*LiΔchrGSK3::LbGSK3*) parasite infectivity in macrophages at 4 and 72 h after infection. Infection was assessed as the percentage of infected macrophages at 4 and 72 h post-infection (**A**) and the number of amastigotes per 100 macrophages (**B**). The results are representative of three independent experiments performed in triplicate. Representative micrographs of THP-1-derived macrophages infected with parasites at 4 and 72 h post-infection (p.i.), stained with rapid panoptic (**C**). Scale bar: 50 µm. Parasite load in mice infected with *Li::Cas9* and *LiΔchrGSK3::LbGSK3*. Parasite burden was quantified by limiting dilution from samples isolated from the liver and spleen of BALB/c (**D**) and IFN-*γ^⁻^*^/⁻^ C57BL/6 mice (**E**). BALB/c and C57BL/6 mice were intravenously inoculated (via tail vein) with 2 × 10^7^ late–log-phase promastigotes from either the *Li::Cas9* or the *LiΔchrGSK3::LbGSK3* lines. Samples were collected on day 20 post-infection. Statistical analysis was performed using analysis of variance followed by Bonferroni’s multiple comparisons test (^*^P < 0.05, ^**^P < 0.01, ^***^P < 0.001).

Infectivity of mutant parasites was evaluated in THP-1-derived macrophages. At 4 h post-infection, *LiΔchrGSK3::LbGSK3* knockdown parasites exhibited reduced infectivity, with a lower (^***^P < 0.001) percentage of infected macrophages (Fig. 6A) and fewer intracellular amastigotes per macrophage (Fig. 6B) compared with *Li::Cas9*. By 72 h post-infection, both parameters were comparable to *Li::Cas9* levels. Representative images of infected THP-1-derived macrophages at 4 and 72 h post-infection are shown in Fig. 6C.

The viability of intracellular amastigotes after macrophage infection was evaluated using a promastigote recovery assay. THP-1-derived macrophages were infected with the different *L. infantum* lines and maintained for 72 h in RPMI medium. At this time point (0 h), no significant differences in intracellular amastigote numbers were observed among the parasite lines analyzed (Fig. S7). Subsequently, infected macrophage cultures were transferred to M199 medium and incubated at 26°C, conditions that favor the differentiation of viable intracellular amastigotes back into proliferative promastigotes. Under these conditions, *Li::Cas9* and *Li::LbGSK3* parasites exhibited progressive promastigote recovery and proliferation throughout the incubation period (48–240 h) (Fig. S7). In contrast, the GSK-3s knockdown parasites (*LiΔchrGSK3::LbGSK3*) displayed a markedly reduced capacity to differentiate into promastigotes and sustain parasite proliferation, with significant reductions in parasite recovery already evident at 48 and 96 h (^#^*P* < 0.05), becoming more pronounced at 144, 192, and 240 h (^####^*P* < 0.0001). These findings suggest that reduced GSK-3s expression compromises parasite viability and/or proliferative fitness following macrophage infection, despite comparable initial intracellular parasite burdens.

Parasite infectivity was further evaluated *in vivo* using BALB/c (Fig. 6D) and IFN*γ*^⁻/⁻^ C57BL/6 (Fig. 6E) mouse models. In both models, mice infected with *LiΔchrGSK3::LbGSK3* exhibited a reduction in splenic parasite burden, with decreases of 1.5-fold (^**^P < 0.01) in BALB/c and 2.5-fold (^***^P < 0.001) in IFN-*γ*^⁻/⁻^ C57BL/6 mice compared with controls. In addition, a 1.75-fold reduction (^*^P < 0.05) in hepatic parasite load was observed in IFN-*γ*^⁻/⁻^ C57BL/6 mice infected with the GSK-3s knockdown parasites.

Promastigote forms of the *L. infantum LiΔchrGSK3::LbGSK3* GSK-3s knockdown line were maintained *in vitro* for more than 20 passages (approximately three months) in M199 medium in the absence of nourseothricin, the selective antibiotic required for maintenance of the episomal plasmid expressing *L. braziliensis* GSK-3s. Despite the absence of selective drug pressure, PCR analysis demonstrated persistent retention of the episomal construct in the parasites. Similarly, parasites recovered from mice experimentally infected with the *LiΔchrGSK3::LbGSK3* line also retained the episomal plasmid. Together, these findings suggest that continuous GSK-3s expression is required for parasite viability, further supporting the essential role of this kinase in *L. infantum.*

## DISCUSSION

Protein kinases play central roles in regulating parasite proliferation, differentiation, stress responses, and host-parasite interactions, and several members of this enzyme family have emerged as promising therapeutic targets in trypanosomatids. In the present study, we provide genetic, phenotypic, and *in vivo* evidence that the short isoform of glycogen synthase kinase 3 (GSK-3s) is essential for *L. infantum* viability and infectivity. Using a CRISPR/Cas9-based genetic strategy with episomal complementation, we show that GSK-3s appears to be required for parasite viability, as complete gene deletion was not achievable, while partial depletion was associated with pronounced alterations in parasite growth, cell-cycle regulation, drug susceptibility, stress tolerance, and infectivity.

Our inability to generate GSK-3s null mutants in the absence of episomal complementation supports the notion that this kinase is likely essential for *L. infantum* viability. Notably, even in the absence of nourseothricin selection, the *LiΔchrGSK3::LbGSK3* knockdown parasites stably retained the episomal *LbGSK3* construct after 20 passages of *in vitro* cultivation, as well as after recovery from experimentally infected mice, further supporting the hypothesis that GSK-3s is essential for the survival of *L. infantum*. Similar findings have been reported in *L. infantum* ascorbate peroxidase knockdown mutants, which retained the episomal plasmid after prolonged passages in the absence of drug pressure, likely to preserve basal protein expression levels necessary for parasite viability (24). This finding aligns with functional studies across kinetoplastids: a systematic screen in *L. mexicana* identified the *GSK3* homolog (*LmxM.18.0270*) as a required gene for promastigotes, further classifying it as essential [Table 1 in Baker and collaborators (11)]. Similarly, studies in *Trypanosoma brucei*, where RNA interference-mediated knockdown of GSK-3s resulted in severe growth arrest and parasite death, validated this enzyme as a drug target in African trypanosomes (16). Complementary pharmacological studies in *L. donovani* demonstrated that indirubin derivatives targeting GSK-3s induce cell-cycle arrest and apoptosis-like death, with phenotypes that could be rescued by GSK-3s overexpression, strongly supporting on-target activity (17). Furthermore, indirubin analogs also demonstrate inhibitory activity against *Trypanosoma cruzi* with good selectivity over mammalian cells (25). Together, these studies in *Leishmania* spp., *T. brucei,* and *T. cruzi,* indicate that GSK-3s performs non-redundant functions that cannot be compensated by the long isoform or by alternative signaling pathways and highlight its potential as a therapeutic target for trypanosomatid diseases.

In mammalian cells, GSK-3 is negatively regulated by the PI3K/AKT/mTOR and Wnt/β-catenin pathways through AKT-mediated phosphorylation or sequestration within multiprotein complexes (26). In *Leishmania*, however, key canonical components of these pathways, including AKT/PKB and β-catenin, have not been identified (27), suggesting that GSK-3s functions within parasite-specific regulatory networks. In mammals, active GSK-3 phosphorylates diverse substrates to control apoptosis, survival signaling, protein synthesis, cell-cycle progression, and stress responses (26). Consistent with a central regulatory role, reduced GSK-3s expression in the *L. infantum LiΔchrGSK3::LbGSK3* line resulted in marked growth impairment, morphological alterations, cell-cycle perturbation, and increased tolerance to oxidative stress. These phenotypes are consistent with the conserved role of GSK-3 in cell-cycle regulation across eukaryotes and with previous findings in trypanosomatids, in which inhibition or depletion of GSK-3s leads to growth arrest and cell-cycle deregulation (15–17). Pharmacological inhibition of GSK-3s in *L. donovani* impairs growth and disrupts cell cycle progression (17). Consistently, GSK-3s depletion in our study caused early accumulation in Sub G0/G1 and subsequent proliferative arrest, providing genetic support for the role of GSK-3s in parasite cell cycle homeostasis and viability.

The morphological abnormalities observed in GSK3-deficient parasites further suggest that this kinase contributes to the maintenance of cytoskeletal organization and cellular integrity, processes that are tightly linked to cell-cycle regulation in kinetoplastids. Similar pleiotropic phenotypes have been reported for other essential kinases in *Leishmania*, including MAPKs and CDKs, reinforcing the idea that kinase signaling networks are critical hubs of parasite fitness and survival (10, 11).

An unexpected yet biologically informative finding of this study was the increased tolerance of GSK-3s knockdown parasites to oxidative stress. In addition, our data demonstrate that antioxidant defense pathways are differentially modulated according to GSK-3s expression levels and oxidative stress conditions. In particular, GSK-3s-deficient parasites displayed selective upregulation of *TXNPx* transcripts both in the presence and absence of H₂O₂, whereas *APX* and *FeSODA* transcript levels were markedly reduced under oxidative stress. In contrast, parasites overexpressing GSK-3s exhibited broader upregulation of antioxidant genes under basal conditions. These findings suggest that modulation of redox-related pathways may contribute to the altered drug response and oxidative stress tolerance phenotypes observed in the mutant parasites. Although GSK-3 has been implicated in redox homeostasis in mammalian systems (26), its role in regulating antioxidant responses in *Leishmania* remains largely unexplored. Classically, GSK-3 is involved in cellular stress responses through modulation of AP-1 signaling, including phosphorylation of Jun family proteins, which contributes to their functional inactivation and regulation of stress- and apoptosis-associated transcriptional programs (26). In contrast, *Leishmania* spp. lack identifiable orthologs of mammalian Jun family genes (JUN, JUNB, and JUND) and canonical AP-1 transcription factors, consistent with the absence of sequence-specific transcription factors and promoter-driven transcriptional regulation in trypanosomatids (27–29). We therefore propose that *Leishmania* GSK-3 regulates stress responses indirectly, through alternative mechanisms such as translational control, unfolded protein response signaling, heat shock pathways, or other post-transcriptional and post-translational regulatory processes that predominate in these parasites (30, 31). Our data suggest that reduced GSK-3s activity elicits compensatory redox adaptations, likely reflecting the highly plastic nature of the trypanothione-dependent antioxidant defense network described in trypanosomatids (32, 33). The observed upregulation of *TXNPx* likely represents an adaptive response that partially mitigates oxidative stress associated with GSK-3s depletion but is insufficient to compensate for the severe defects in growth, cell-cycle progression, and infectivity observed in these parasites. Conversely, parasites overexpressing exogenous GSK-3s (*Li::LbGSK3*) displayed increased transcript levels of multiple antioxidant enzymes, including *APX*, *TXNPx*, and *FeSODA*. In contrast to the selective response observed upon GSK-3s depletion, this broader induction of antioxidant genes is consistent with a more global redox adjustment associated with elevated GSK-3s activity. Resistance to oxidative stress is a critical determinant of parasite survival within the hostile intracellular environment of host macrophages, where reactive oxygen and nitrogen species constitute major effector mechanisms of parasite killing. Previous studies have shown that increased *TXNPx* expression is associated with enhanced oxidative stress tolerance, infectivity, and resistance to antileishmanial drugs in *Leishmania* spp. (34–36).

GSK-3s knockdown parasites exhibited altered susceptibility to antileishmanial drugs, including increased resistance to Sb^III^ and miltefosine. Drug resistance in *Leishmania* is typically multifactorial and associated with metabolic remodeling, stress-adaptive responses, and changes in membrane dynamics rather than direct target modification. In this context, the enhanced resistance observed in GSK-3s knockdown parasites likely reflects a broader adaptive state induced by disruption of a central signaling kinase (37). Increased oxidative stress tolerance and upregulation of tryparedoxin peroxidase are consistent with reduced Sb^III^ sensitivity, given the reliance of antimonials on redox imbalance for parasite killing (32, 33, 38). Together, these findings suggest that altered drug susceptibility in GSK-3s knockdown parasites arises from indirect, adaptive responses to global signaling perturbation rather than from classical resistance mechanisms.

Notably, the striking 111-fold resistance to the GSK-3s inhibitor 6-bromo-5-methylindirubin-3′-oxime (6-BIO) provides strong genetic confirmation that the GSK-3s parasite is the primary intracellular target of this compound in *L. infantum*. This finding is fully consistent with earlier biochemical, structural, and pharmacological studies demonstrating selective inhibition of leishmanial GSK-3s by indirubin derivatives (12, 15, 17). The concordance between genetic depletion and pharmacological resistance strengthens the validation of GSK-3s as a bona fide drug target.

GSK-3s knockdown parasites exhibited reduced infectivity in macrophages at early time points (4 h post-infection), consistent with an impaired ability to withstand host-derived oxidative stress induced by rapid NRF2-dependent macrophage responses (39). In addition, these mutant parasites also displayed a markedly reduced capacity to differentiate back into proliferative promastigotes and sustain parasite growth following macrophage infection, further supporting a defect in parasite viability and proliferative fitness after the intracellular stage. Importantly, GSK-3s knockdown parasites exhibited significantly lower parasite burdens in both BALB/c and IFN-*γ*^⁻/⁻^C57BL/6 mouse models, demonstrating that GSK-3s is essential for parasite survival and infectivity in the mammalian host. This attenuated phenotype likely reflects combined defects in parasite proliferation, stress adaptation, and early adaptation to the intracellular macrophage environment. Consistent with this interpretation, disruption of kinase signaling pathways has been widely associated with reduced *Leishmania* infectivity (10, 11, 19). Genetic disruption of other infectivity-associated genes similarly leads to reduced parasite loads in target organs; for example, *L. donovani* p27-null parasites display markedly reduced splenic and hepatic burdens in infected BALB/c mice (40). In line with these observations, *LiΔchrGSK3::LbGSK3* parasites showed a 1.5-fold to 2.5-fold reduction in splenic burden and an approximately 1.75-fold reduction in hepatic burden, reinforcing the interpretation that reduced GSK-3s expression may compromise the establishment and maintenance of visceral infection.

Bioinformatic and structural analyses comparing parasite and human GSK-3 active sites have revealed exploitable differences that may enable the development of inhibitors with enhanced selectivity for the parasitic enzyme, thereby minimizing potential host toxicity (10, 41). Pharmacological inhibition of parasite GSK-3 disrupts cell-cycle regulation, leading to cell-cycle arrest and parasite death, underscoring the essential role of this kinase in pathways required for parasite proliferation (17, 41). In parallel, modulation of host GSK-3β has emerged as a complementary therapeutic strategy: activation of GSK-3β in *L. donovani*-infected macrophages promotes a proinflammatory cytokine profile, characterized by increased IL-12 and reduced IL-10 production, thereby restricting intracellular parasite survival (42). Moreover, recent reviews have positioned GSK-3s as a central regulatory node in trypanosomatid biology and a target of growing pharmacological interest (41). The genetic essentiality of GSK-3s demonstrated here, together with its observed impact on parasite infectivity, supports the potential development of GSK-3-directed therapies, either as standalone interventions or in combination with existing antileishmanial drugs.

In conclusion, this study provides genetic evidence suggesting that GSK-3s is required for optimal viability of *L. infantum* and plays an important role in regulating parasite growth, stress adaptation, drug response, and infectivity. By integrating genetic, cellular, and *in vivo* approaches, our work contributes to the biological validation of GSK-3s as a therapeutic target and provides a framework for future studies aimed at exploring this kinase for the development of new and more effective treatments for visceral leishmaniasis.

## MATERIALS AND METHODS

### Cultivation and maintenance of parasites

Promastigote forms of *L. infantum* (MHOM/BR/1974/PP75), originally obtained from the IRR/Fiocruz Minas, were cultured in GIBCO M199 medium (Gibco, Thermo Fisher Scientific, Waltham, MA, USA) supplemented with 10% fetal bovine serum (Gibco), 5 μg/mL hemin (Sigma-Aldrich, St. Louis, MO, USA), and 5 μM biopterin (pH 7.0) (Sigma-Aldrich). Cultures were maintained at 26°C, and parasite densities were determined using a Neubauer hemocytometer. Parasites were subcultured twice weekly by inoculating 1 × 10^6^ cells into 5 mL of fresh medium. Unless stated otherwise, all experiments were performed using promastigotes in the logarithmic phase of growth.

### Transfections

The initial attempt to disrupt the single-copy GSK-3s gene (LINF_180007700) in *L. infantum* using the CRISPR/Cas9 system was performed following the methodology described in a previous study (8). The *L. infantum* line expresses *Sp*Cas9 and T7 RNA polymerase from the pTB007 plasmid (*Li::Cas9*), which carries hygromycin resistance (20), as well as the red fluorescent protein *tdTomato*, which confers resistance to blasticidin (21). This *Li::Cas9 (tdTomato)* line was transfected with donor DNA cassettes and single-guide RNA (sgRNA) templates using the LeishGEdit toolkit (http://www.leishgedit.net/) (8). Plasmids pTNeo v1 and pTPuro v1, conferring resistance to neomycin (NEO) and puromycin (Puro), respectively, served as templates for PCR amplification of donor DNA fragments containing 30 bp homology arms using primers A and B (Table S1). sgRNA templates targeting the 5′UTR and 3′UTR of GSK-3s were generated by PCR with primer sets C–E and D–E, respectively (Table S1).

For the second strategy to generate endogenous *L. infantum* GSK3-deficient mutants, the *Li::Cas9* line was transfected with the pIR1SAT_LbGSK3 construct, which contains the *Streptothricin acetyltransferase* (SAT) gene conferring resistance to nourseothricin (Sigma-Aldrich). The plasmid was assembled using Gibson Assembly (NEB New England Biolabs, Ipswich, MA, EUA) with primers F and G (Table S1). The insert sequence corresponded to *L. braziliensis* GSK-3s (LBRM2903_180007900), which was cloned into *Bgl*II-linearized pIR1_SAT in *Escherichia coli* TOP10F’ (Invitrogen, Waltham, MA, USA). Bacterial transformation was performed by incubation at 4°C for 30 min followed by heat shock at 42°C for 45 s. Transformed cells were recovered at 37°C for 1 h to induce ampicillin resistance gene expression and subsequently plated on *Luria-Bertani* agar supplemented with ampicillin (Sigma-Aldrich). The construct was sequenced using the Sanger method (Genewiz, Azenta Life Sciences, South Plainfield, NJ, USA), with M13 primers. Contigs were assembled using DNASTAR software, and sequences were analyzed using MultAlin software (http://multalin.toulouse.inra.fr/multalin/). Transfection of *Li::Cas9* promastigotes with the pIR1SAT_LbGSK3 plasmid was carried out as previously described (43), and recombinant parasites were selected using 100 μg/mL nourseothricin sulfate (Sigma-Aldrich).

Next, *Li::LbGSK3* parasites were transfected with donor DNA cassettes and sgRNA templates to disrupt the chromosomal copy of GSK-3s, generating the *Li::ΔchrGSK3:: LbGSK3* line. Donor DNA fragments were amplified from pTNeo v1 and pTPuro v1 using primers A and B, while sgRNA templates targeting the 5′UTR and 3′UTR were generated using primer pairs C–E and D–E (Table S1). All transfections were performed according to the protocol described in a previous study (43). Selection of *Leishmania* was conducted in M199 medium supplemented with selective antibiotics corresponding to each resistance marker: 10 µg/mL blasticidin (Gibco), 40 μg/mL G418-neomycin (Gibco), 400 μg/mL hygromycin B (Invitrogen), 100 μg/mL nourseothricin (Sigma-Aldrich), and 30 μg/mL puromycin (Gibco). After initial selection, antibiotics were maintained only during weekly passages; however, all phenotypic assays were performed in the absence of drugs. Genomic DNA was extracted using DNAzol reagent (Thermo Fisher Scientific, Waltham, MA, USA), following the manufacturer’s instructions. Gene deletion was assessed by PCR using primers targeting the GSK-3s coding sequence (primers H and I) and primers designed to verify replacement of GSK-3s alleles by antibiotic resistance markers (primers J to M) (Table S1).

### Global sequence alignment

The sequence analysis was performed using tBLASTn. Global alignment of the amino acid sequences of the long GSK-3 isoforms from *L. mexicana* (LmxM.22.0490) and *L. infantum* (LINF_220010400), as well as the short (GSK-3s, LINF_180007700) and long (GSK-3l, LINF_220010400) isoforms from *L. infantum,* was performed using MAFFT software (version 7.505). In addition, the amino acid sequences of GSK-3s from *L. braziliensis* (LBRM2903_180007900) and *L. infantum* (LINF_180007700) were also aligned using MAFFT. Sequence identity and similarity were determined from the resulting alignments using the EMBOSS infoalign tool. Domain architecture analysis was carried out based on InterProScan annotations (EMBL-EBI), enabling the identification of the glycogen synthase kinase-3 domain (IPR050591), the protein kinase domain (IPR000719), and the serine/threonine protein kinase active site (IPR008271).

To identify sequences potentially homologous to the long GSK3l isoform of *T. brucei*, similarity searches were performed using the tBLASTn algorithm implemented in the NCBI BLAST + package (v2.12) and *hmmsearch* from the HMMER suite (v3.4). Candidate sequences were ranked based on similarity scores. Protein domain validation was conducted using InterProScan (v5.72) to detect the conserved glycogen synthase kinase-3 functional domain (IPR050591). Multiple sequence alignments were generated to assess conservation of major functional domains (Fig. S1).

### RT-qPCR

To investigate transcript levels, quantitative reverse-transcription PCR (RT-qPCR) analysis was performed using cDNA from mutants and *Li::Cas9* parasites. Promastigotes (approximately 1  ×  10^8^) were resuspended in 1 mL of TRIzol Reagent (Invitrogen), and total RNA was extracted using the chloroform method. The RNA was treated with a Turbo DNA-free Kit (Invitrogen) according to the manufacturer’s instructions, and complementary DNA (cDNA) was obtained using Superscript II reverse transcriptase (Invitrogen) following the manufacturer’s instructions. All cDNA samples were diluted to 100 ng/μL and used in the RT-qPCR amplification reaction using Power SYBR Green Master Mix (Applied Biosystems, Thermo Fisher Scientific, Waltham, MA, USA). The specific primers are listed in Table S1 (primers N to C.1). The levels of transcripts of the following enzymes were evaluated: GSK-3s from *L. infantum* (LINF_180007700) and *L. braziliensis* (LBRM2903_180007900); ascorbate peroxidase, APX (LINF_340005600); tryparedoxin peroxidase, TXNPx (LINF_150018600, LINF_150018800, and LINF_150019000); iron superoxide dismutase A, FeSODA (LINF_080007900); and Putative Protein Kinase, GSK-3l (LINF_220010400). Relative quantification of the target genes in the mutants was compared with that in the *Li::Cas9* parasite background using the constitutive DNA polymerase gene (LINF_160021500) as a normalizer. Amplifications were performed using the QuantStudioTM 12 Flex system (Thermo Fisher Scientific), following the standard cycling of the machine. Data were analyzed using the comparative CT method (2^-ΔΔCT^). All reactions were conducted in triplicate on a 96-well plate. The amplification process involved an initial cycle of 50°C for 2 min and 95°C for 10 min, followed by 40 cycles of 95°C for 15 s and 60°C for 1 min each, and concluded with a final cycle of 95°C for 15 s, 60°C for 15 s, and 95°C for 15 s. We performed three independent biological replicates for each parasite line, with each replicate conducted in triplicate.

### Growth curve

To assess parasite growth, control and mutant promastigotes were inoculated at an initial density of 1 × 10⁵ cells/mL in M-199 medium (Gibco) and maintained at 26°C. Parasite density was determined daily over an 8-day period using a Neubauer hemocytometer under a Laborlux S microscope (Leitz, Wetzlar, Germany). Three independent experiments were performed, each in triplicate.

### Promastigotes morphological analysis

Promastigotes from control and mutant strains of *L. infantum* were harvested at 48 h and 96 h post-passage and washed twice with phosphate-buffered saline (PBS, pH 7.4). Parasite suspensions were then applied to glass slides, air-dried, and fixed with methanol for 5 minutes. Fixed slides were stained with 10% Giemsa solution (Sigma-Aldrich) for 15–20 min, rinsed gently with distilled water, and air-dried. Parasite morphology was evaluated under a light microscope (Axio Observer.A1, Zeiss, Germany) at 63× magnification using oil immersion, and images were captured using a digital camera attached to the microscope.

Morphometric analysis was performed to evaluate cell body size and flagellum length. Measurements were performed using ImageJ software (National Institutes of Health, Bethesda, MD, USA) after calibration with the scale bar. Cell body diameter was determined by the maximum width, and flagellum length was measured using the segmented line tool. For each condition, 50 parasites were analyzed per sample from three independent experiments, and data were expressed as mean ± standard deviation (SD).

### Cell cycle analysis

Promastigotes (1 × 10^6^ parasites) of *L. infantum* (control and mutants) were harvested during growth periods of 48 and 96 h, washed twice withPBS (pH 7.4), and fixed in 70% ethanol at 4°C overnight. Fixed cells were washed with PBS and incubated for 30 min at room temperature in the dark with a staining solution containing propidium iodide (PI, 50 μg/mL; Sigma-Aldrich) and RNase A (100 μg/mL; Sigma-Aldrich) to simultaneously stain DNA and remove RNA (44). Cell cycle distribution was analyzed using a BD FACSCalibur flow cytometer (BD Biosciences, Franklin Lakes, NJ, USA), acquiring 30,000 events per sample. Data were processed using FlowJo software (v10), and the percentage of cells in sub-G0/G1, G0/G1, S, and G2/M phases was determined. Experiments were performed in three independent biological replicates.

### EC_50_ assays

To evaluate parasite susceptibility to trivalent antimony (Sb^III^) (Sigma-Aldrich), hydrogen peroxide (H₂O₂) (Sigma-Aldrich), miltefosine (Cayman Chemical, Ann Arbor, MI, USA), amphotericin B (Laboratorios Richet, Buenos Aires, Argentina), and 6-bromo-5-methylindirubin-3′-oxime (6-BIO) (Sigma-Aldrich), 2 × 10⁶ promastigotes were incubated in 1 mL of M199 medium (Gibco) containing increasing concentrations of each compound. The concentration ranges tested for promastigotes of the *Li::Cas9*, *Li::LbGSK3*, and *LiΔchrGSK3::LbGSK3* lines were as follows: Sb^III^, 6.25–800 μM; amphotericin B, 0.0125–0.8 μM; miltefosine, 5–640 μM; H₂O₂, 12.5–2,500 μM; and 6-BIO, 0.0625–20 μM. Parasite growth in the presence or absence of the compounds was evaluated after 48 h of incubation by measuring fluorescence emission in black, clear-bottom 96-well plates (Corning Inc., Corning, NY, USA), using excitation and emission wavelengths of 554 nm and 581 nm, respectively, on a Varioskan LUX plate reader (Thermo Fisher Scientific, Waltham, MA, USA). The half-maximal effective concentration (EC_50_) was determined from three independent experiments performed in triplicate. EC_50_ values were estimated by nonlinear regression using a variable-slope model fitted to the “log(inhibitor) *versus* response” equation.

The *in vitro* activity against intracellular amastigotes of *L. infantum* was evaluated using THP-1-derived macrophages infected with the *tdTomato*-expressing PP75 strain, as previously described (21). THP-1 cells were differentiated with 50 ng/mL phorbol 12-myristate 13-acetate (PMA) and infected with stationary-phase promastigotes at a ratio of 20 parasites per macrophage for 4 h. After removal of non-internalized parasites, infected macrophages were incubated for 72 h in the presence of increasing concentrations of Sb^III^ (0.625–100 μM), miltefosine (1.5–240 μM), or H₂O₂ (6.25–100 μM). Intracellular amastigote growth was quantified by fluorescence measurement after 72 h of compound exposure using a Varioskan LUX microplate reader (Thermo Fisher Scientific), with excitation and emission wavelengths set at 554 nm and 581 nm, respectively. The concentration required to inhibit 50% of parasite growth (EC_50_) was determined based on the reduction in fluorescence relative to untreated controls. EC_50_ values were calculated by nonlinear regression using a variable-slope model (“log[inhibitor] *versus* response”) in GraphPad Prism v8.2.0 (GraphPad Software, CA, USA).

### THP1- Macrophages infection

To assess the infectivity capacity of mutant parasites, THP-1 cells were cultured in complete RPMI-1640 medium (supplemented with 10% fetal bovine serum, 100 U/mL of penicillin, and 100 μg/mL of streptomycin—all from Gibco) in a 5% CO_2_ incubator at 37°C. Human monocytic THP-1 cells were differentiated into macrophages by adding 50 ng/mL of PMA (Sigma-Aldrich). After 72 h, the macrophages were infected with stationary-phase promastigotes of *L. infantum* (20 parasites per macrophage) for 4 h. Parasites that failed to infect the cells were removed by washing, and the infected macrophages were incubated in RPMI-1640 medium for 72 h. Infectivity was assessed immediately after 4 and 72 h of incubation. Coverslips were stained with rapid panoptic (Laborclin Produtos para Laboratórios Ltda., Pinhais, PR, Brazil) and photographed. Infection was quantified by counting intracellular amastigotes and the percentage of infected macrophages, using ImageJ software, based on the counting of 100 macrophages per replicate for each sample. Three independent experiments were performed in triplicate.

To evaluate the viability of intracellular amastigotes after macrophage infection, we adapted a protocol described in a previous study (45). THP-1-derived macrophages were infected with the different *L. infantum* lines in RPMI medium and maintained for 3 days (72 h) at 37°C in a humidified atmosphere containing 5% CO_2_. After this incubation period, the culture medium was replaced with M199 medium supplemented with 10% fetal calf serum, and the plates were incubated at 26°C for 10 days (0–240 h) to allow the differentiation of viable intracellular amastigotes into promastigotes. Promastigote growth was monitored every 48 h by counting parasites in culture supernatants using a Neubauer hemocytometer after homogenization of the cultures. The results were expressed as the mean number of promastigotes per mL of M199 medium ± SD from three independent experiments.

### *In vivo* infection assays

For the in *vivo* infection assays, male BALB/c (*Mus musculus*) and IFN-*γ*^−/−^ C57BL/6 mice (25 g; 4–5 weeks old) were used. Animals were housed in the Experimental Animal Facility of IRR-Fiocruz under standard conditions, in clean cages with free access to water and commercial chow. To evaluate the infectivity of mutant parasites, groups of BALB/c and IFN-*γ*^−/−^ C57BL/6 mice (*n* = 4 per group) were intravenously inoculated (via tail vein) with 2 × 10^7^ late-log-phase promastigotes from either the *Li::Cas9* or the *LiΔchrGSK3::LbGSK3* lines. After 20 days of infection, the animals were euthanized. Parasite loads in liver and spleen tissues were quantified using a limiting dilution assay. Briefly, organs were collected, and tissue fragments were homogenized using an Ultra-Turrax disperser (IKA-Werke GmbH & Co. KG., Staufen, Germany) in 1 mL of M-199 medium. The resulting homogenates were serially diluted (10-fold) in 96-well microtiter plates and incubated at 26°C for 10 days. Wells containing motile promastigotes were identified using an inverted microscope (Axiovert 25; Carl Zeiss, Oberkochen, Germany), and parasite burden was calculated based on the highest dilution at which parasite growth was observed after incubation.

### Statistical analysis

All experiments were conducted in triplicate, and data were analyzed using GraphPad Prism 8.0 (GraphPad Software Inc., San Diego, CA, USA). One-way analysis of variance (ANOVA) or two-way ANOVA were used to assess overall differences between groups, followed by the Bonferroni post hoc test to compare the mutants with the *Li::Cas9* or the *Li::LbGSK3*. Statistical significance was set as *P* < 0.05. *P*-values were reported following the format of GraphPad Prism 8.0 (GraphPad Software Inc.), where ns (*P* > 0.05), ^*^(*P* ≤ 0.05), ^**^(*P* ≤ 0.01), ^***^(*P* ≤ 0.001), ^****^(*P* ≤ 0.0001).

## SUPPLEMENTAL MATERIAL

Supplemental material aac.00247-26-s0001.pdf Fig. S1 to S7; Table S1.

ASM does not own the copyrights to Supplemental Material that may be linked to, or accessed through, an article. The authors have granted ASM a non-exclusive, world-wide license to publish the Supplemental Material files. Please contact the corresponding author directly for reuse.
