# Supplementary material for: Essential role of glycogen synthase kinase 3 in regulating growth, drug response, and infectivity in Leishmania infantum
Source: Antimicrob Agents Chemother. 2026 Jul 6;70(8):e00247-26. doi: 10.1128/aac.00247-26 (PMC13435798; doi:10.1128/aac.00247-26)
Supplement: Supplementary file 1 [file aac.00247-26-s0001.pdf]

SUPPLEMENTAL MATERIAL

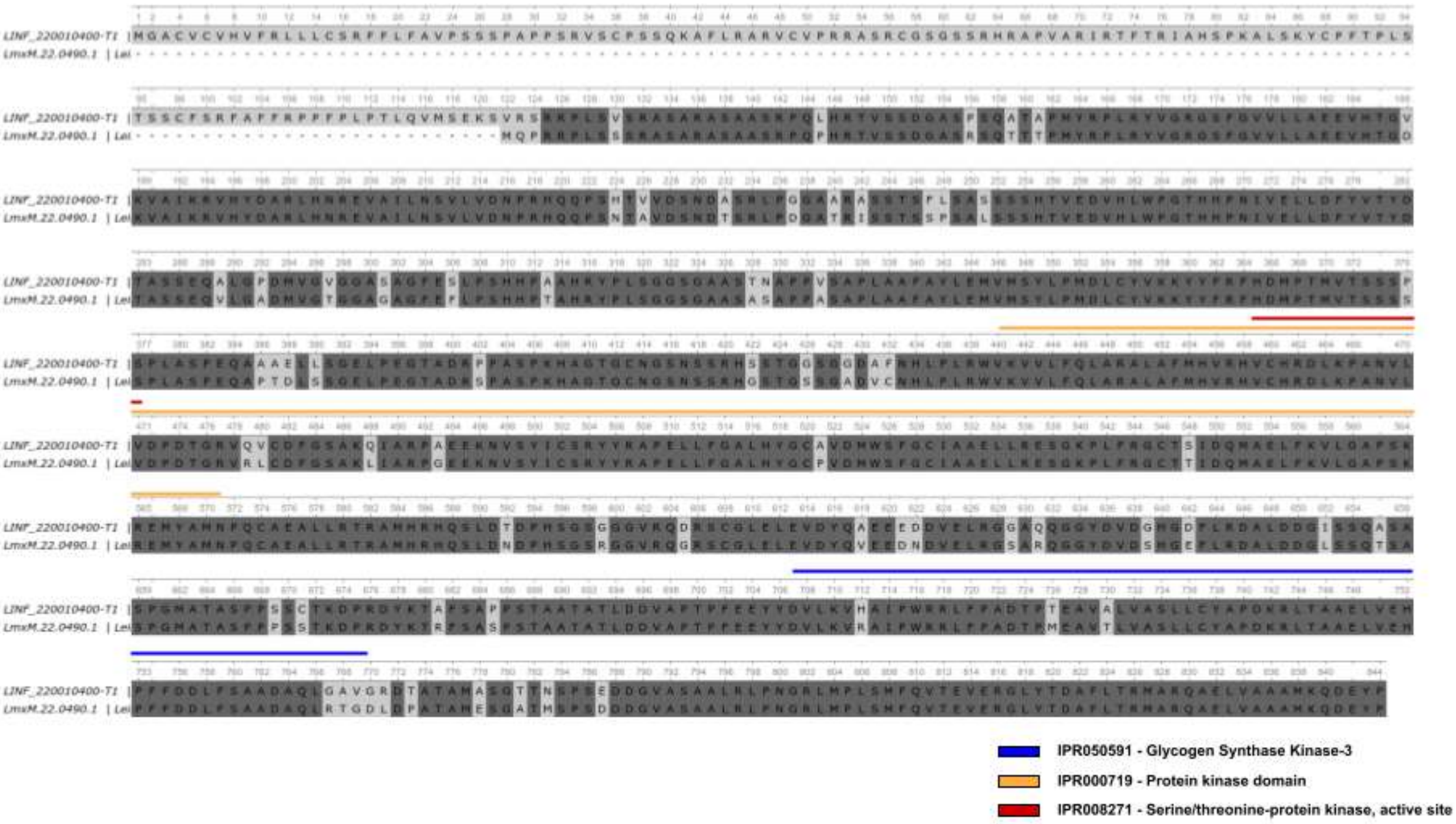

FIG S1 Global sequence alignment of the long GSK-3 isoforms (GSK-3l) from *L. mexicana* (*LmxM.22.0490*) and *L. infantum* (*LINF_220010400*). The *L. mexicana* GSK-3l consists of 723 amino acids, whereas the *L. infantum* GSK-3l contains 844 amino acids. The alignment was performed using MAFFT, revealing 89.9% sequence identity and 91.8% similarity between the sequences. Domain architecture analysis identified the following domains: Glycogen Synthase Kinase-3 (InterPro: IPR050591), highlighted in blue; the protein kinase domain (InterPro: IPR000719), in yellow; and the serine/threonine protein kinase active site (InterPro: IPR008271), in red.

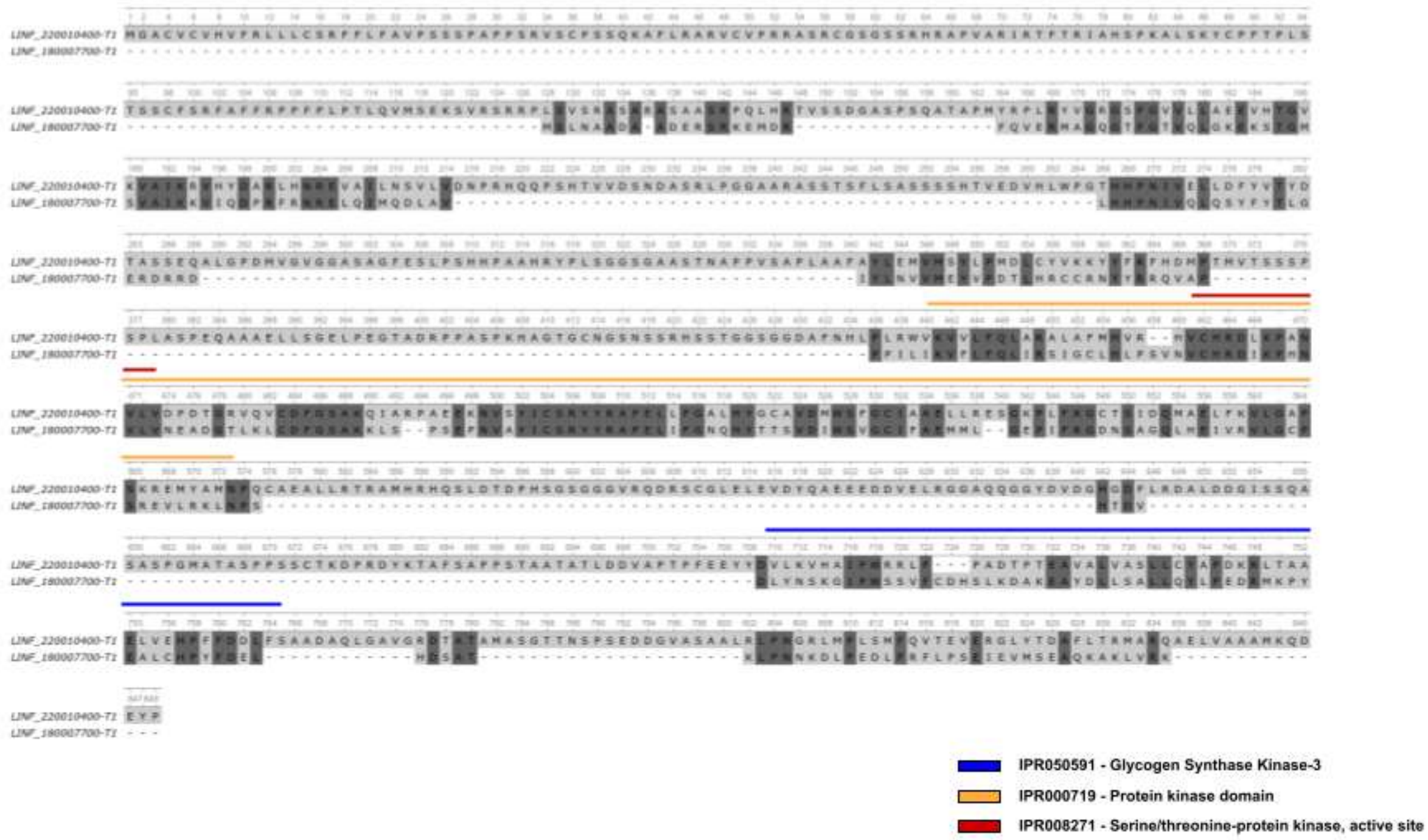

FIG S2 Global sequence alignment between the short (GSK-3s, *LINF_180007700*) and long (GSK-3l, *LINF_220010400*) isoforms of GSK-3 from *L. infantum*. The alignment was performed using MAFFT, revealing 43.6% sequence identity and 63.9% similarity between the two isoforms. Domain architecture analysis identified the following domains: Glycogen Synthase Kinase-3 (InterPro: IPR050591), highlighted in blue; the protein kinase domain (InterPro: IPR000719), in yellow; and the serine/threonine protein kinase active site (InterPro: IPR008271), in red.

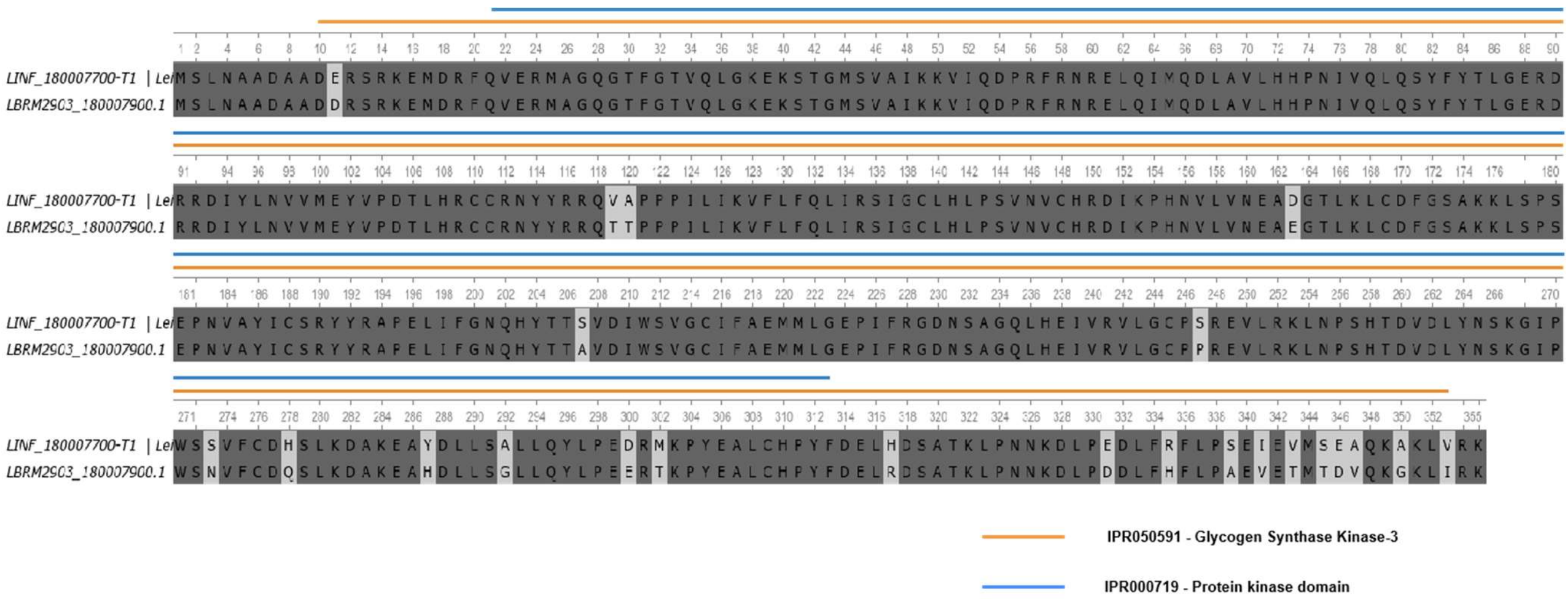

FIG S3 Global alignment of the GSK-3s amino acid sequences from *L. braziliensis* (*LBRM2903_180007900*) and *L. infantum (LINF_180007700*) generated using MAFFT. The alignment comprises 355 amino acids and shows a high degree of conservation between the two sequences, with 93.5% identity and 96.9% similarity. The protein sequence is highlighted in red, while the functional (catalytic) domain is indicated in blue.

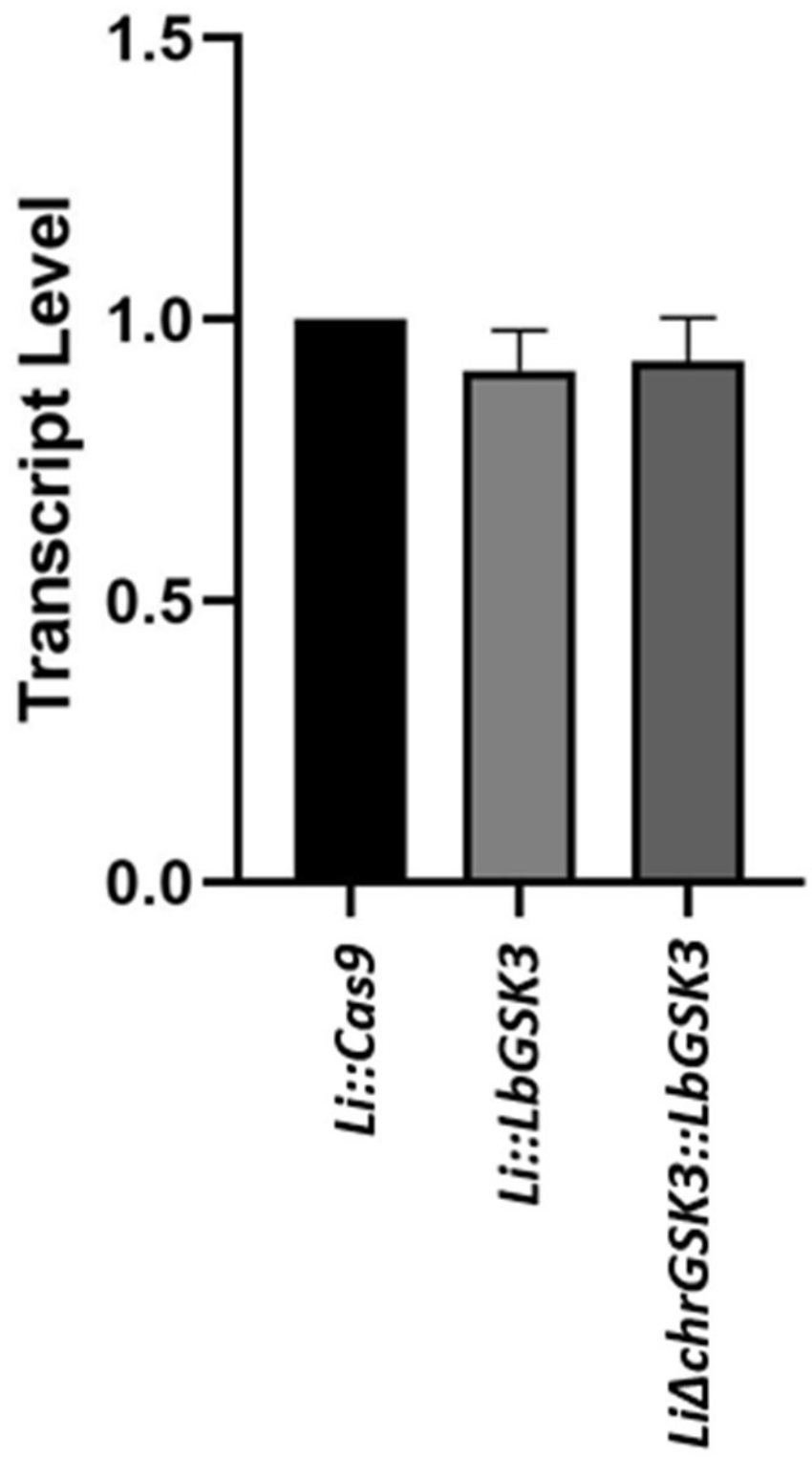

FIG S4 Transcriptional analysis of the long GSK-3 isoform (*LINF_220010400*) in *L. infantum* mutant parasites. Transcript levels of the long *LiGSK-3* isoform (*LiGSK-3l*) were evaluated by RT-qPCR in *Li::Cas9* and mutant promastigotes using the B1 and C1 primer pair listed in Additional file 1: Table S1. The DNA polymerase gene (*LINF_160021500*) was used as the constitutive endogenous control, and relative transcript levels were calculated using the $2^{-\Delta\Delta CT}$ comparative method. Statistical analysis was performed using one-way ANOVA followed by Dunnett's post hoc test to compare mutant lines with the *Li::Cas9* control group.

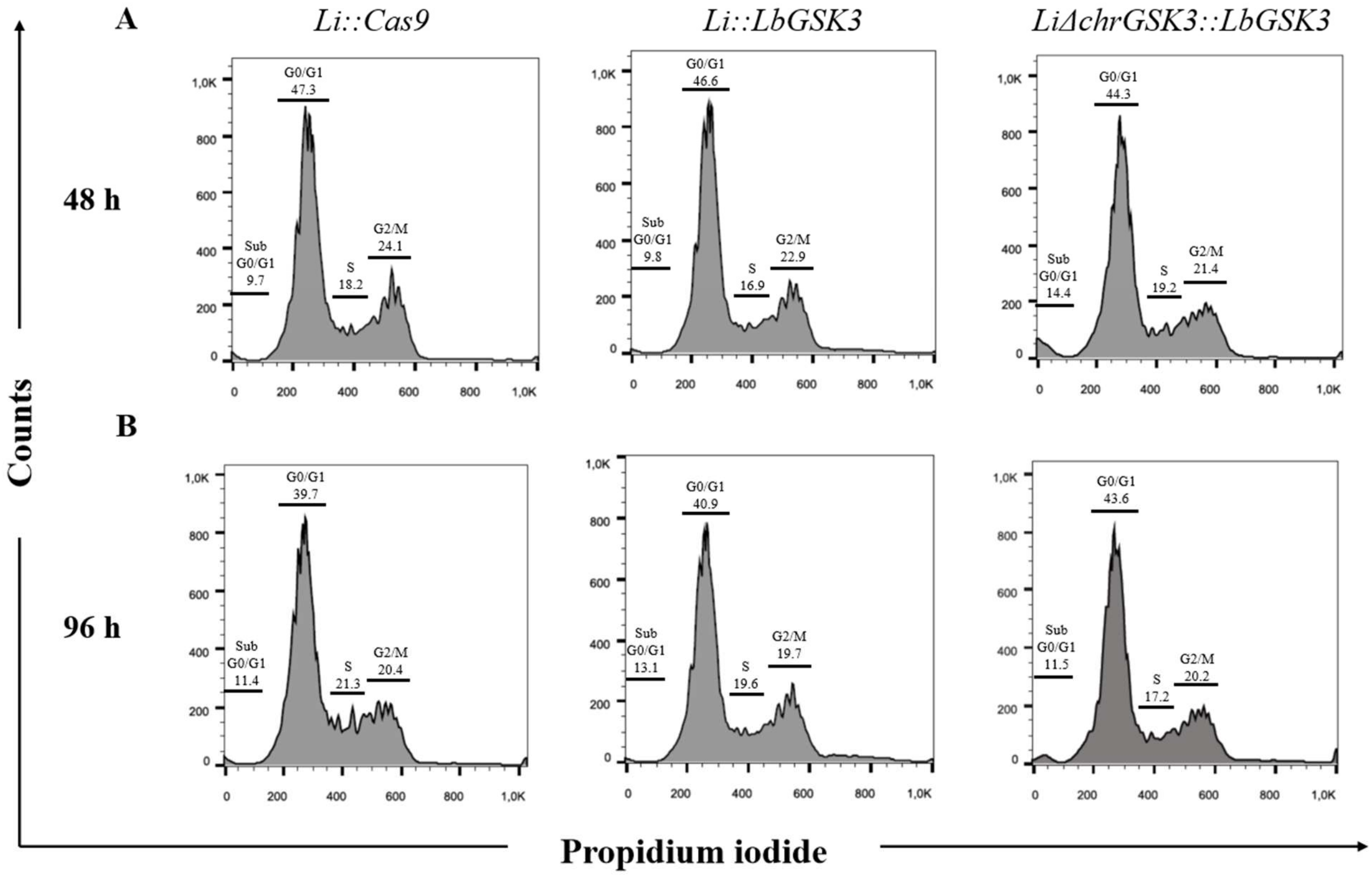

FIG S5 Representative histograms of cell cycle analysis in GSK-3s mutant *L. infantum* parasites. Cell cycle profiles of *Li::Cas9*, *Li::LbGSK3*, and *LiΔchrGSK3::LbGSK3* promastigotes were analyzed at (A) 48 h and (B) 96 h of growth. Parasites were stained with propidium iodide, and DNA content was evaluated by flow cytometry.

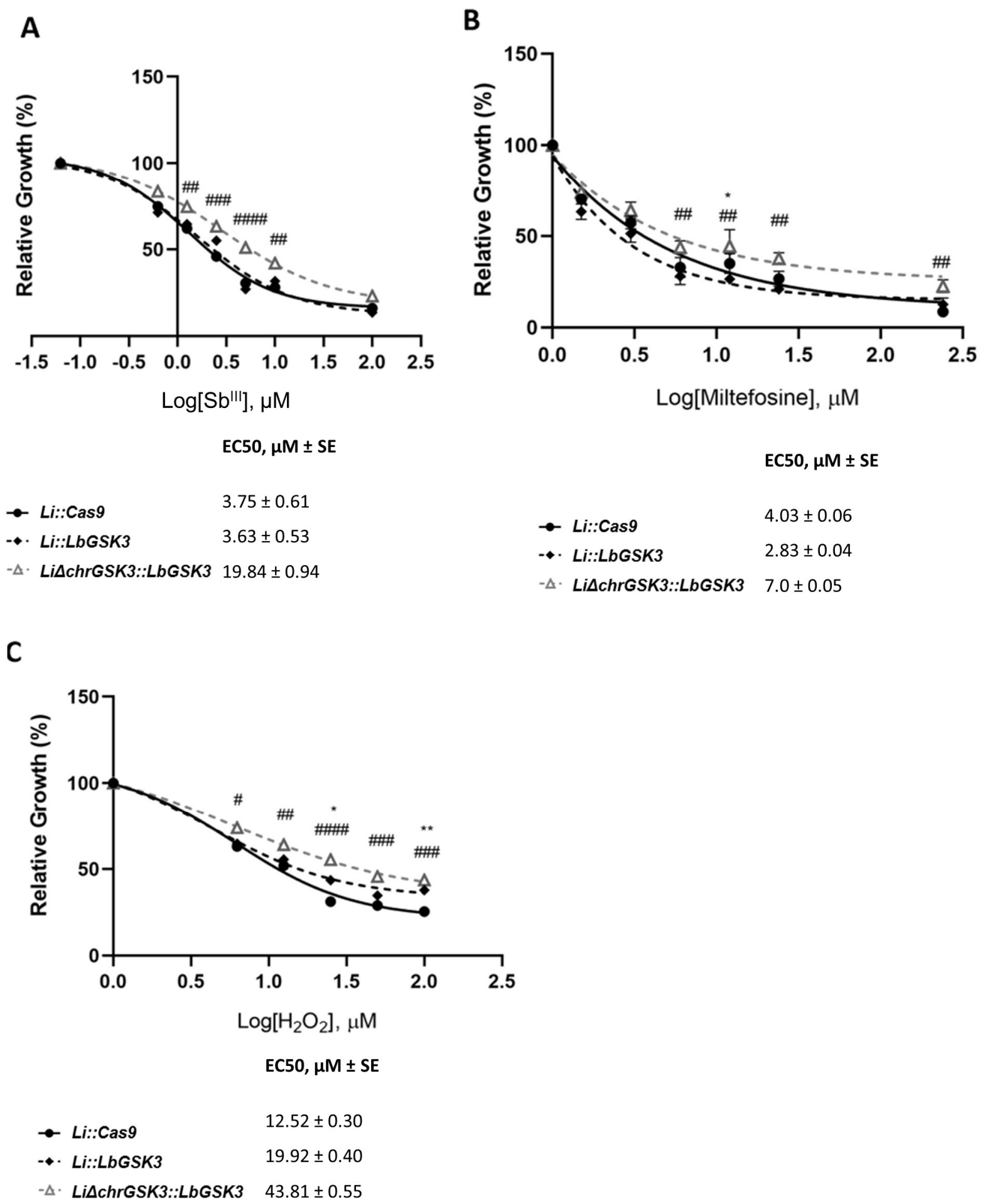

FIG S6 Drug susceptibility profiles of *L. infantum* intracellular amastigotes expressing altered GSK-3s levels. *Li::Cas9* and GSK-3s mutant parasites were cultured in the presence of different concentrations of (A) Sb^{III} (0.625–100 μM), (B) miltefosine (1.5–240 μM) and (C) H_2O_2 (6.25– 100 μM). Intracellular amastigote growth was determined after 72 h of incubation by measuring fluorescence using excitation and emission wavelengths of 554 and 581 nm, respectively. Dose–response curves represent the mean ± standard deviation (SD) of three independent experiments performed in triplicate. The EC_{50} was determined using a non-linear regression–variable slope model with the 'log (inhibitor) *versus* response' equation in GraphPad Prism. Two-way ANOVA with Dunnett post hoc test was used to compare *Li::Cas9* parasites and mutants for each drug concentration; $^{*}p < 0.05$, $^{**}p < 0.01$ for *Li::Cas9 vs. Li::LbGSK3*; $^{\#\#}p < 0.01$, $^{\#\#\#}p < 0.001$, and $^{\#\#\#\#}p < 0.0001$ for *Li::Cas9 vs. LiΔchrGSK3::LbGSK3*.

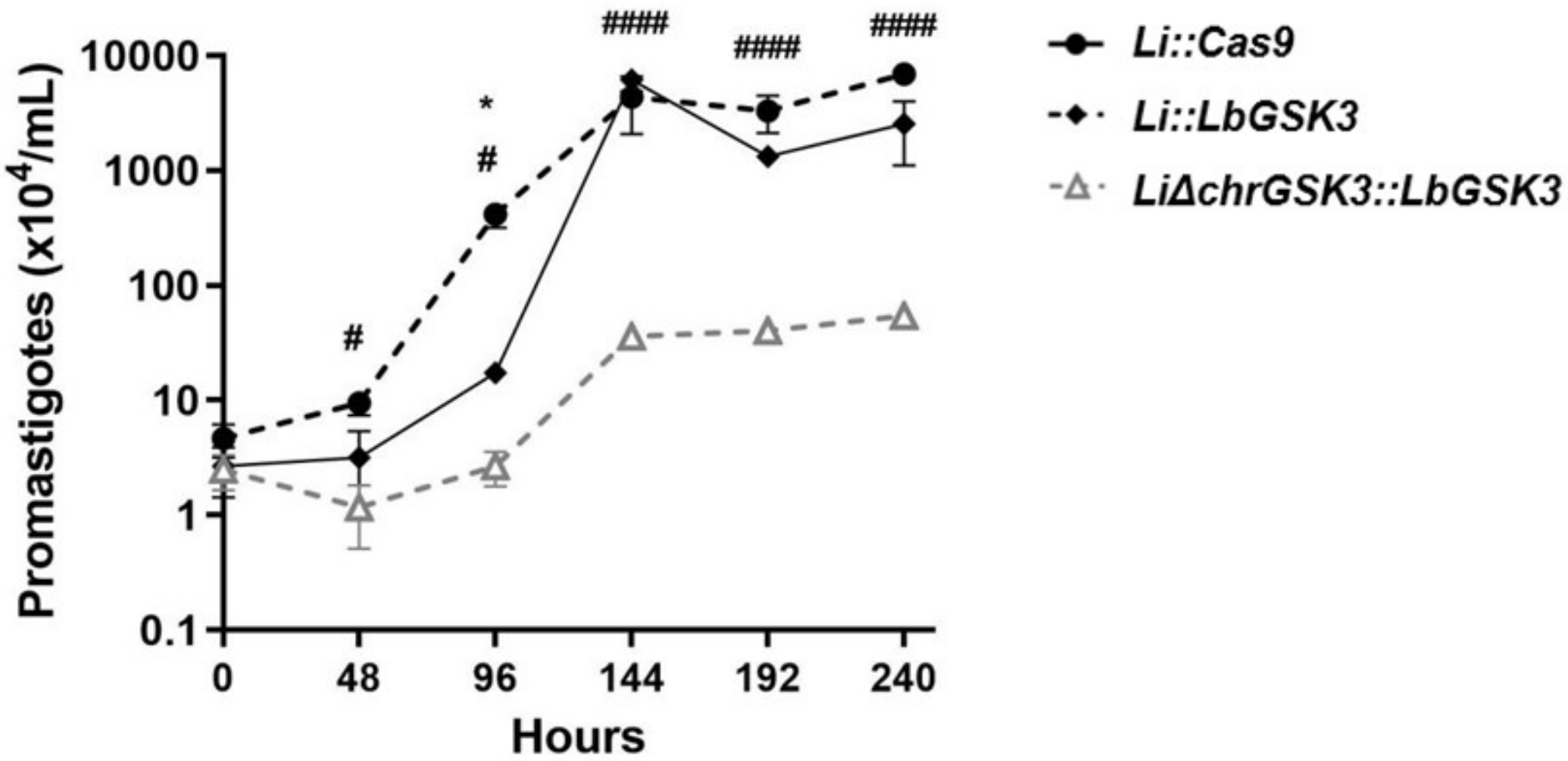

FIG S7 Evaluation of intracellular amastigote viability by promastigote recovery assay. THP-1–derived macrophages were infected with the different *L. infantum* lines in RPMI medium and maintained at 37 °C with 5% CO_2 for 72 h. After this period, the culture medium was replaced with M199 medium, and the cultures were incubated at 26 °C for 10 days (0 – 240 h) to allow the differentiation of viable intracellular amastigotes into promastigotes. Promastigote growth was assessed every 48 h by counting parasites recovered from the culture supernatants using a Neubauer hemocytometer. Results were expressed as the mean number of promastigotes per mL ± standard deviation (SD) from three independent experiments. One-way ANOVA with Dunnett's post hoc test was used to compare control parasites and mutants. *Li::Cas9* was used as the control. $^{*}p < 0.05$ for *Li::Cas9 vs. Li::LbGSK3*; $^{\#}p < 0.05$ and $^{\#\#\#\#}p < 0.0001$ for *Li::Cas9 vs. LiΔchrGSK3::LbGSK3*.

Table S1 List of primers used in this study

		PRIMER NAME	PRIMER SEQUENCE 5' → 3'	TM (°C)	FRAGMENT (bp)
Donor DNA	**A**	Upstream.FW	AGGCGGAGAAGCAAAGAAATGGAAGGGGAGgtataatgcagacctgctgc	60	pTNEO 1757
	**B**	Downstream.RV	TGACATGACGCTTCCCCTCTGTCATCCTTTccaatttgagagacctgtgc		pTPURO 1854
sgRNA	**C**	5'sgRNA	gaaattaatacgactcactataggGTAGTGGGAACACACAAGTAgttttagagctagaaatagc	60	164
	**D**	3'sgRNA	gaaattaatacgactcactataggGTTTCTTCGGGCCCGCTGCAgttttagagctagaaatagc		
	**E**	G00.rv	aaaagcaccgactcggtgccactttttcaagttgataacggactagccttattttaacttgctatttctagctctaaaac		
Gibson Assembly	**F**	SAT.GSK3. FW	CCTCGCTGCCCGCGTCCGGAGATCTCCACCATGTCGCTCAACG	60	1123
	**G**	SAT.GSK3.RV	AGTACATCACAAGACTCATAGATCTTTACTTGCGGATTAGCTTGC		
GSK3	**H**	LiGSK3.FW	ATGTCGCTCAACGCTGC	60	1068
	**I**	LiGSK3.RV	CTACTTGCGAACCAGCT		
Integration PCR	**J**	5'UTR.GSK3Li.FW	CTTCCAGGGTGTTTCTGA	60	GSK3 1373
	**K**	3'UTR.LiGSK3.RV	GCTGACATGACGCTTCCC		PURO 1437
	**L**	NEO.RV	GCCAACGCTATGTCCTGATA		NEO 449
	**M**	PURO.RV	GGCTTACGTGTCATGCACCAT		
qPCR	**N**	qPCR.LiGSK3.FW	ATCGCATGAAGCCTTACGAA	63	127
	**O**	qPCR.LiGSK3.RV	TCGATCTCGCTCGGCAGG		
	**P**	qPCR.LbGSK3.FW	CGACGTCAAACCACCCCG	62	198
	**Q**	qPCR.LbGSK3.RV	CTCCGACGGTGACAACTTC		

	**R**	qPCR.GSK3.LiLb.FW	CGTGTGCCACCGCGACATCA	63	123
	**S**	qPCR.GSK3.LiLb.RV	ATGTATGCCACGTTCGGCTC		
	**T**	rt.DNApol.fw	CGAGGGCAAGACATAC	62	69
	**U**	rt.DNApol.rv	GAGAGCGGGCACCAATCAC		
	**V**	qPCR.APX.FW	CATCAAATTCTCCGGCTACCA	62	113
	**W**	qPCR.APX.RV	TCTCAACCTTCGGATTCAAGAC		
	**X**	qPCR.FeSODA. FW	GAGCACGCCTACTACAAAGA	62	111
	**Y**	qPCR.FeSODA. RV	CTTCGTGGCCTTCTCATACA		
	**Z**	qPCR.TXNPx.FW	TCGCTTCAACGAGCTCAAC	62	
	**A.1**	qPCR.TXNPx.RV	TCGGCCAGCATTGGAATC		129
	**B.1**	qPCR.GSK.L.FW	GGCAAGTGGAACGACAAATTC	62	
	**C.1**	qPCR.GSK.L.RV	TCCGTGACCTGGAACATAGA		108
